# Traditionally Used Medicinal Plants of Armenia

**DOI:** 10.3390/plants13233411

**Published:** 2024-12-04

**Authors:** Arpine Ayvazyan, Christian Zidorn

**Affiliations:** 1 Pharmazeutisches Institut, Abteilung Pharmazeutische Biologie, Christian-Albrechts-Universität zu Kiel, Gutenbergstraße 76, 24118 Kiel, Germany; 2 Division of Pharmaceutical Biotechnology, Department of Pharmaceutical Biology and Biotechnology, Wroclaw Medical University, Borowska 211, 50-556 Wrocław, Poland

**Keywords:** medicinal plants, traditional medicine, ethnopharmacology, Caucasus, Armenia

## Abstract

The rich and diverse flora of Armenia has been used for medicinal purposes for at least 3000 years. The relevant literature in Armenian, English, and Russian revealed a vast array of used medicinal plants, some of them unique to the Caucasus region. The usage of medicinal plants confirms the position of Armenia as a country at the crossroads of Asia and Europe because of its traditional usage of medicinal plants from both continents. Literature data in Armenian, English, and Russian on medicinal plants of Armenia were mainly obtained using various electronic databases. From all available sources, 320 Armenian medicinal plant species were extracted with their botanical and local names and traditional uses. The use of medicinal plants by the Armenian people is systematically compiled, including the used plant organs and preparations and the ailments for which the various taxa are/were used. Medicinal plants of Armenia are represented for both wild and cultivated species. Some of the taxa used are unique to Armenia or the Caucasus region, while many other species are also used in various other countries. Some of the species from traditional Armenian medicine are currently being studied using modern methods.

## 1. Introduction

**Figure 1:**
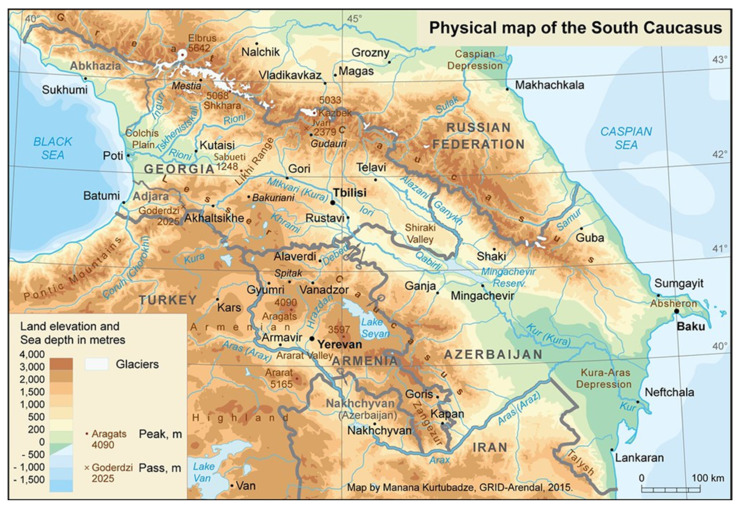
Physical map of the South Caucasus (Cartographer: GRIDArendal/Manana Kurtubadze; https://www.grida.no/resources/7628 (accessed on 1 December 2024)).

Armenia is situated in Western Asia, in the Transcaucasus (Southern Caucasus) region, to the north-east of the Armenian Highlands [1]. The country is landlocked; it is about 145 km from the Black Sea and 175 km from the Caspian Sea. Armenia borders Georgia, Azerbaijan, Turkey, and Iran (Figure 1) [2]. Armenia is a small country covering an area of about 29,800 km^2^, which is almost the size of Belgium (about 30,700 km^2^). According to the Human Development Report [3], Armenia belongs to the countries with a high human development index. Politically, Armenia is subdivided into 11 administrative units, including 10 provinces called marzes (մարզեր; Aragatsotn, Ararat, Armavir, Gegharkunik, Kotayk, Lori, Shirak, Syunik, Tavush, and Vayots Dzor) and the capital city, Yerevan [4]. Armenia is a mountainous country with the lowest point at 375 m above sea level and the highest point at 4090 m above sea level (Mount Aragats) and an average elevation of 1850 m [2].

The climate in Armenia is represented by dry and cold winters, due to air masses from the Siberian high-pressure system in winter, and warm and dry summers, due to the Arabian subtropical high pressure in the west and the Asian depression in the east [5]. Despite its relatively small area, Armenia has a diverse vegetation characterized by the Caucasian mixed forest and Eastern Anatolian Mountain steppe ecoregions [6], among which steppes predominate, and only 8% are forests [7].

**Figure 2:**
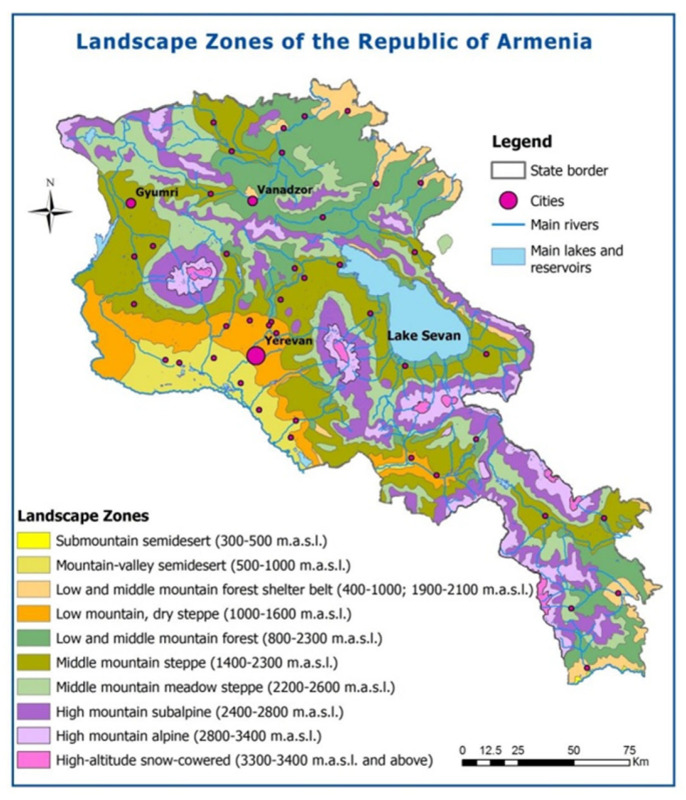
Landscape zones of the Republic of Armenia (The fifth national report to convention on biological diversity, 2014).

The vegetation of Armenia has a pronounced altitudinal zonation, and its richness and distribution are mainly determined by the mountainous relief [8]. According to the national atlas of Armenia [9], there are ten landscape zones in Armenia (Figure 2). Additionally, there are a number of intrazonal ecosystems (marshes, rocks, screes) that are present in almost all altitudinal zones [8].

The diversity of landscapes is an important factor determining the diversity of vegetation in Armenia [2].

- The lower mountain belts (480–1200 m a.s.l.) are covered by semi-desert vegetation dominated by *Artemisia fragrans* Willd. [synonym: *A. erivanica* (Bess.) Grossh.] (Asteraceae) [2].
- The middle mountain belts (1200–1800 m a.s.l.) are covered by steppes or sparse arid woodland. Steppes are mainly dominated by *Stipa* species (Poaceae) [5]; 18 species of *Stipa* are known in Armenia. Sparse arid woodland is composed of *Pistacia atlantica* var. *mutica* Rech.f. (Anacardiaceae), *Amygdalus fenzliana* Fritsch (Rosaceae), and *Rhamnus pallasii* Fisch. and C.A.Mey. (Rhamnaceae) [5].
- The upper mountain belts (1900–2300 m a.s.l.) are covered with meadow steppes and shrub steppes [5]. Meadow steppes composed primarily of taxa from the Poaceae, including species of *Festuca*, *Stipa*, *Bromus*, *Elymus*, and *Koeleria*, along with wild relatives of cereals (*Secale montanum* Guss., *Hordeum violaceum* Boiss. and Hohen.) [10].
- The altitudinal forest belt ranges from 500 to 1500 (2000) m, depending on the region, and can approach 2400 m, when open park stands are included [2].
- The subalpine mountains (2300–2900 m a.s.l.) are covered with subalpine meadows mainly dominated by *Festuca varia* Haenke [5] and include mixed-steppes forests comprised of *Quercus macranthera* Fisch. and C.A.Mey. (Fagaceae) forests, *Acer trautvetteri* Medw. (Sapindaceae), and *Betula litwinowii* Doluch. (Betulaceae) [10].
- The alpine mountains (2700–3700 m a.s.l.) are covered with alpine meadows mainly dominated by the taxa from the Poaceae family (e.g., *Poa alpina* L.). In addition, *Taraxacum stevenii* DC. (Asteraceae), *Alchemilla* spp. (Rosaceae), *Potentilla* spp. (Rosaceae), *Primula* spp. (Primulaceae), *Geranium* spp. (Geraniaceae), *Campanula* spp. (Campanulaceae), and *Pedicularis* spp. (Orobanchaceae) [5], *Kobresia* and *Carex* grasslands (Cyperaceae), *Rhododendron caucasium* Pall. (Ericaceae), and *Carum caucasicum* (M.Bieb.) Boiss. (Apiaceae) are occurring in the mountains, including the highest peaks of Mount Aragats [10].

Armenia is thus characterized by a rich and diverse flora with more than 3000 plant species [11,12,13,14,15,16,17,18,19,20,21,22,23]. Throughout the country, there are both ubiquitous and endemic plant species; from both groups, various organs have been used in folk medicine since ancient times [24]. The presence of several biogeographical zones within the country gives rise to a total of 123 endemic plant species [25], of which a huge number are Rosaceae (31 species), Asteraceae (27 species), Fabaceae (15 species), and Poaceae (13 species) [26]. Moreover, many species are used as food by the local population [24]. There are about 200 species of edible plants, which are used both in fresh and in processed form (boiled, pickles, etc.), making up to 10–15% of the food consumed. *Falcaria* (Apiaceae), *Hippomarathrum* (Apiaceae), *Asparagus* (Asparagaceae), and *Chaerophyllum* (Apiaceae) are some of the most widely used genera. In addition, Armenia is home to about 120 species of wild fruits and berries, as well as more than 350 species contributing to the production of honey; these are widely spread throughout the country [8]. On the other hand, there are about 450 plant species in the Red Book of Armenia, including some endemics [27]. In order to preserve rare, endangered, endemic species and ensure their reproduction in the natural environment, unique ecosystems and special protected areas of nature have been defined in Armenia: four national parks (“Sevan”, “Dilijan”, “Lake Arpi”, and “Arevik”), three state reserves (“Khosrov Forest”, “Erebuni”, and “Shikahokh”), and 27 sanctuaries, which together cover 10.3% of the territory of Armenia [28].

The main purpose of this review is to summarize the use of ethnomedicinal plants in Armenia. The core is comprised of a list of wild and cultivated medicinal plants currently or formerly used in Armenia (Table S1), indicating their local names and describing the centuries-old history of their use in folk medicine. The article also discusses the current state of the study of medicinal plants of Armenia.

## 2. Materials and Methods

The search of scientific literature on the use of plants in the traditional medicine of Armenia available in international journals was conducted using Google Scholar, Web of Science, PubMed, and Science Direct using the keywords “traditional medicine”, “medicinal plants”, “ethnopharmacology”, “Armenia”, “Armenian medicinal plants”, “vegetation of Armenia”, “Armenian traditional medicine”, and “history of Armenian traditional medicine”. Taking into account the fact that the search was limited to literature published in English and many works on medicinal plants of Armenia are published in Armenian and/or Russian, the search was also performed in these languages using “Հայաստանի դեղաբույսեր (Hayastani deghabuyser)”, “Հայկական ժողովրդական բժշկություն (Haykakan zhoghovrdakan bzhshkutyun)”, “Էնդեմիկ բույսեր (Endemik h)”, Հայաստանի Կարմիր գիրք (Hayastani Karmir girk)”, “Ուտելի բույսեր (Uteli buyser)”, “Лeкapcтвeнныe pacтeния Apмeнии (Lekarstvennyye rasteniya Armenii)”, and “Иcтopия мeдицины в Apмeнии (Istoriya meditsiny v Armenii)” as keywords. Many papers have only been published in national journals, which complicated the search process. Therefore, the websites “Proceedings of the YSU B: Chemical and Biological Sciences” (https://journals.ysu.am/index.php/proceedings-chem-biol (accessed on 1 October 2021)), “Collection of Scientific Articles of YSU SSS” (http://www.old.ysu.am/ssspub/en/1656325614 (accessed on 3 October 2021)), and “Biological Journal of Armenia” (https://www2.flib.sci.am/journal/Biology/ (accessed on 1 October 2021)) were used. The keywords “plant”, “medicinal plant”, and “flora of Armenia” were used for the search on the websites of Proceedings of the YSU B: Chemical and Biological Sciences (selected collections published from 2009 to 2022), and Biological Journal of Armenia (selected collections published from 2006 to 2023). On the website of the Collection of Scientific Articles of YSU SSS, due to the lack of search, all issues of collections published from 2012 to 2022 were opened one by one, with particular attention to the section Natural and Physical–Mathematical Sciences. Articles corresponding to the topic by title and content were preselected and screened. Papers containing only studies on animals were excluded from the present study, and for papers containing both animal experiments and other studies (e.g., determination of antioxidant or antimicrobial activity), the animal part was again excluded from selection. Often the botanical name with the addition of “Armenia” or “Caucasus” and/or the local name of the plant species (e.g., “*Crataegus armena* Pojark. Armenia” or “Հայկական ալոճ (Haykakan aloch)”) was used for searches. This method was used mainly to search for endemic plants of the Caucasus and/or Armenia. Hardcopies of the books with descriptions of the names of medicinal plants in Armenia and their use in folk medicine were lent from Armenian libraries, such as the National Library of Armenia, the Sarkis and Marie Izmirlian Library of Yerevan State University, and the Mesrop Mashtots Institute of Ancient Manuscripts (Yerevan Matenadaran). Since plant names in books are sometimes presented only in local names or in Russian, the Armenian–Latin–Russian–Anglo–French-–German Dictionary of Plant Names (Ghazaryan, 1981) was used to search the botanical names of the plants (http://www.nayiri.com/imagedDictionaryBrowser.jsp?dictionaryId=81 (accessed on 10 October 2021)) [29]. The exact local and botanical names of plant species, as well as plant species endemic to the Caucasus and/or Armenia, were checked against the books of Takhtajan (“Flora of Armenia” (1958–2009). The botanical names of the plants were also checked against World Flora Online (http://www.worldfloraonline.org/ (accessed on 10 October 2021)). The plant species included in the Red Book of Plants of the Republic of Armenia (RA) are sourced according to the RA Government Decree No. 72-N of 2010 (https://www.arlis.am/ (accessed on 21 September 2023)), and cultivated plants species according to the RA Ministry of Economy Decree No. 20-L of 2023 (https://mineconomy.am/page/1330 (accessed on 25 November 2023)). Confusing, unclear, or erroneous data on plant part, botanical identity, or mode of use were excluded from the review. Information on medicinal plants characteristics, timing of collection, use with other plant species, and phytochemical composition was also excluded.

## 3. Results and Discussion

### 3.1. History of Traditional Medicine in Armenia

From the rich flora of Armenia, about 800 species could be used as medicinal plants [30]. The history of Armenian traditional medicine goes back about 3000 years [31]. The writings of Amirdovlat Amasiaci (1420–1496) are still considered the basis for the study of Armenian phytotherapy and Armenian folk medicine [32]. The work presented a study on the medieval physician Amirdovlat Amasiaci, who lived and practiced in Anatolia in the 15th century. The study examines Amasiaci’s works on medicine written in Middle Armenian, the colloquial Armenian language of the time. The books are mainly devoted to phytotherapy and pharmacology using medicinal plants, animal products, and minerals [32]. Finding a place in the ancient pharmacopoeias, the names of medicinal plants and their uses were also included in the works of ancient scientists. Herodotus (484–425 B.C.), Strabo (63 B.C.–24 A.D.), Xenophon (430–354 B.C.), and Tacitus (56–120 A.D.) all mentioned the natural riches of Armenia, and the medicinal and edible plants in particular. Many Armenian wines, beer, sesame oil, almond oil, and the resin of the turpentine tree (*Pistacia terebinthus* L.) are mentioned in Xenophon’s “Anabasis” work [33]. Pliny the Elder (24–79 A.D.) writes in his “Naturalis Historia” that the plant laserwort (*Laserpitium* L.) was known in Armenia and Marastan, which Roman patricians used to restore lost health and youth [33]. The names of many wild medicinal plants, such as *Lychnis*, *Bryonia*, and *Nigella*, can also be found in the works of Armenian historians. According to the Armenian historian Movses Khorenatsi (410–490 A.D.), the Old-World sycamore (*Platanus orientalis* L.) of the Armenian flora, whose leaves and bark were used to treat skin diseases, including leprosy, was considered a sacred tree, and was an object of worship [33].

### 3.2. Checklist of Armenian Medicinal Plants

In this review, we have listed a total of 320 medicinal plant species belonging to 231 genera and 79 families from all available sources. A detailed list of plants used in Armenian traditional medicine with their botanical and local names, plant families, parts used, and traditional uses in alphabetical order is presented in Supplementary Material Table S1. The form of use is mostly the whole plant or parts of it, in the form of decoctions, infusions, broths or saps, and, in some cases, teas prepared from the plants. Often, other ingredients (alcohol, wine, honey, oil) are added. The solvents used for the preparation of the decoctions or infusions are mainly water, alcohol, and water-alcohol or water-ether mixtures.

**Figure 3:**
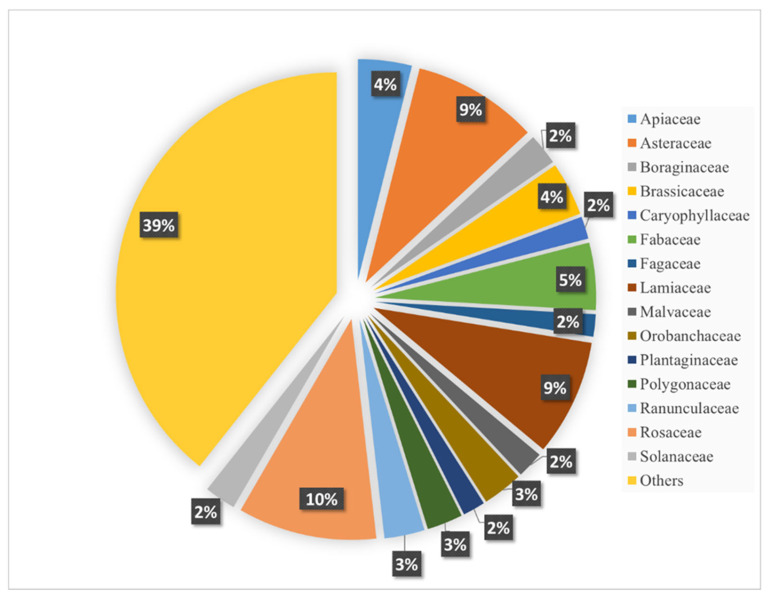
Percentage representation of plant families used in Armenian traditional medicine. The “Others” includes all families represented by four or fewer plant species.

The dominant family are the Rosaceae with 31 species (Figure 3). Significantly, the family also tops the list of herbs used in Pakistan [34], Montenegro [35], Bulgaria, and Italy [36]. Asteraceae and Lamiaceae are the next two families with a high number of species (28 and 26 species, respectively) used in Armenian traditional medicine (Figure 3). The family Asteraceae is one of the most popular plant families in the world, with over 25,000 species and about 1700 genera. Their use in folk medicine is diverse and multifaceted [37]. For instance, the genus *Baccharis* is known for its plant species with anti-rheumatic, antispasmodic, digestive, anti-inflammatory, and other properties [38]. On the other hand, many species belonging to the genus *Pterocaulon* Elliott are widely used to treat respiratory, skin, liver, and many other diseases [39]. The family Lamiaceae is no less widespread and used in folk medicine than the family Asteraceae [40], numbering over 6500 species and about 230 genera, among which nearly 900 species are medicinal plants [41,42,43].

The Asteraceae and Lamiaceae are followed by Fabaceae (15 species), Apiaceae and Brassicaceae (12 species, each), Orobanchaceae and Ranunculaceae (nine species, each), Polygonaceae (eight species), Boraginaceae and Solanaceae (seven species, each), Malvaceae (six species), and Caryophyllaceae, Fagaceae and Plantaginaceae (five species, each; Figure 3).

**Figure 4:**
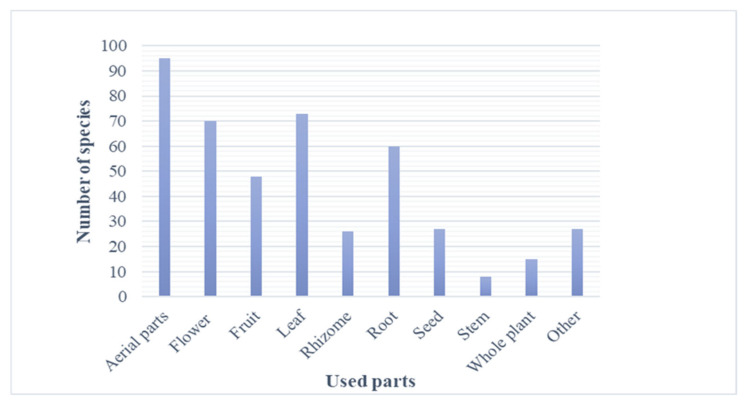
List of the most frequently used plant parts together with the number of relevant species used in Armenian traditional medicine.

Various parts of the plant have been used for medicinal purposes in Armenian traditional medicine. As shown in Figure 4, the aerial parts of the plants were used mostly, followed by leaves, flowers, roots, fruits, seeds, rhizomes, and stems.

### 3.3. Medicinally Important Plant Genera Used in Armenia

Medicinal plants account for about 10% of the species composition of the flora of Armenia. In this review, a total of 231 genera are presented that are used in Armenian traditional medicine. The genus *Artemisia* with about 300 species is widely used in traditional medicine [44]. There are 16 species of Artemisia occurring in Armenia, of which *A. abrotanum* L., *A. absinthium* L., and *A. dracunculus* L․ are used as appetizers, diuretics, and laxatives, as well as to counter anemia, fever, rheumatism, headaches, and to counter eye and bone pains (Table S1) [45,46].

Since ancient times, numerous species belonging to the genera *Berberis*, *Crataegus*, *Hypericum*, *Juniperus*, *Rhamnus*, and *Rosa* have been used in folk medicine in Armenia [2].

In Armenia, the genus *Crataegus* is presented by 23 species [47], of which six species are listed in the Red Book of Plants of RA (RA Government Decree N 72-N of 2010), and three species are presented in this review as medicinal plants (Table S1). The species *C. pontica* K.Koch, *C. orientalis* (Mill.) M.Bieb., and *C. atrosanguinea* Pojark. are recommended for use as food [47]. Another large genus is *Rosa*, with 31 species growing in Armenia [9], among which four are medicinally used species (Table S1). Currently, fresh and dried fruits and petals of *R. canina* L., *R. corymbifera* Borkh., and *R. spinosissima* L. are sold in the markets of Yerevan for culinary and medicinal purposes [48]. In Armenian traditional medicine, a decoction of roots of *R. canina* is used in the treatment of haemorrhoids in a bath․ In addition, the juice of the fruit is used to counter diseases of the eyes and ears, as well as colds, stomach, and intestinal diseases [41], while *R. corymbifera* and *R. spinosissima* are better known for fighting liver and heart disease [45,49]. The oil obtained from these plant species is used in cosmetics [48].

The genus *Hypericum* is represented in Armenia by 39 species, while *Berberis*-by 33 species [13], but only three species from each genus are mentioned for medicinal use (Table S1). *Hypericum perforatum* L. is one of the most commonly sold species in Yerevan markets as a medicinal remedy [48] for gingivitis, neuralgia, malaria, and several female diseases, as well as for rheumatism, scabies, and gout [45]. Between medicinally used and food plant species, there is often a strong overlap, e.g., when the same species used mainly for tea or food is also used for medicinal and prophylactic purposes [50]. Good examples of plants used both for food and medicinal purposes are *Berberis orientalis* C.K. Schneid. and *B. vulgaris* L., fresh and dried fruits of which are sold in Yerevan markets for making jam and liqueur and to use as a spice and tea [48]. In folk medicine, root extracts are used to counter intestinal obstruction, and crushed roots and fruits are used to counter tuberculosis [49,51]. Allegedly, more than 160 plant species have been sold in the markets of Yerevan as food and medicine or as food species with presumed medicinal value, as ornamental plants, as sources of wood, or as insect repellents. These species belong to 44; of these Asteraceae, Rosaceae, and Apiaceae contribute the highest number of species, while of the 110 genera, *Tragopogon* and *Crataegus* are the most species rich [48].

Other ethnomedicinally important genera found in Armenia are *Astragalus* (a total of 125 spp.), *Carex* (101 spp.), *Veronica* (86 spp.), *Verbascum* (67 spp.), *Orobanche* (65 spp.), *Orchis* (58 spp.), *Trifolium* (47 spp.), *Centaurea* (46 spp.), *Nepeta* (46 spp.), *Potentilla* (46 spp.), *Allium* (44 spp.), *Galium* (44 spp.), *Senecio* (43 spp.), *Salvia* (41 spp.), *Taraxacum* (33 spp.), *Salix* (31 spp.), *Lathyrus* (30 spp.), *Geranium* (30 spp.), *Myosotis* (29 spp.), *Gentiana* (28 spp.), *Linaria* (28 spp.), *Alchemilla* (28 spp.), *Cuscuta* (27 spp.), *Trigonella* (25 spp.), *Pyrus* (24 spp.), *Plantago* (23 spp.), *Sedum* (21 spp.), *Echinops* (20 spp.), *Inula* (20 spp), *Prunus* (18 spp.), *Amygdalus* (15 spp.), *Viola* (14 spp.), *Leontodon* (13 spp.), *Primula* (13 spp.), *Rubus* (13 spp.), *Cotoneaster* (13 spp.), *Ribes* (12 spp.), *Asperula* (12 spp.), *Anchusa* (11 spp.), *Solanum* (11 spp.), *Brassica* (10 spp.), and *Dracocephalum* (10 spp.) [11,12,13,14,15,16,17,18,19].

### 3.4. Ethnomedicinal Plant Species Used in Armenian Traditional Medicine

In Table S1, we present 320 species of medicinal plants used in Armenia, among which there are plant species that are also well known in other countries. Among the species presented in this study, a significant number of species are included in the European Pharmacopoeia.

*Achillea millefolium* L. is widely used in traditional European medicine to treat inflammatory and spasmodic diseases of the gastrointestinal tract and hepato-biliary disorders and as an appetite enhancer. In addition, it is also used externally to counter skin inflammation and wound healing [52,53,54,55]. In the traditional medicine of Armenia, the plant is used to counter diseases of the upper respiratory tract and dysentery and as a styptic to counter uterine bleeding [51,56]. The taxon is classified as non-poisonous and is authorized for use in alcoholic beverages by the Food and Drug Administration. However, harmful effects have been reported after its use in humans and in animal tests [57]. Some results have shown that long-term use of *A. millefolium* extract in high doses may cause toxicity [58]. Dalsenter et al. (2004) investigated the effect of an aqueous extract of *A. millefolium* leaves at different doses on the fertility of Wistar rats. These authors concluded that there was no long-term reproductive toxicological risk associated with doses of *A. millefolium* commonly consumed by humans [59]. The rhizome of *Acorus calamus* L. is used in traditional medicine. In Armenia, the plant is used as an appetizer, analgesic, astringent, and disinfectant [45,51]. Similarly, the *A. calamus* is also used in Bulgaria [60]. In Poland, the plant is used internally as an expectorant, anti-inflammatory, and in the treatment of some gastrointestinal diseases, as well as externally to counter hair loss [61]. The whole plant *Cichorium intybus* L. is used for medicinal purposes, but the roots are mainly used. Even in ancient Egypt and Rome, the plant was known as a digestive aid [62]. The resin obtained from the plant (formed by burning the plant in dishes) is used by Caucasian peoples to treat a number of skin diseases (old wounds, ulcers, boils) [51,63]․ In Armenian folk medicine, the sap of the plant is drunk to prevent blocked veins, improve appetite, stimulate digestion, and as a diuretic [51]. *Chelidonium majus* L. has been known as a medicinal plant since the time of the ancient Romans [64]. The aerial parts of the plant are used for medicinal purposes, and sometimes, so are the roots. In Armenian traditional medicine, the plant is used to counter arthritis, headaches, and especially migraines [65]. In Germany, the whole plant is used in the form of tea and decoction, as an analgesic, and as an antispasmodic agent to counter liver and gallbladder diseases [64]. In Poland, *C. majus* is used as an anti-inflammatory, analgesic, and anthelminthic agent, as well as to counter gastrointestinal diseases [66]. Nevertheless, some researchers have reported adverse effects on both animals and humans. *C. majus* latex has been reported to cause severe irritation of the mucous membranes of the mouth, throat, stomach, and intestines when swallowed. When applied directly to the skin, the plant causes irritation and allergic contact dermatitis [67]. According to several investigations, toxic liver damage may develop after the use of *C. majus* extract or herbal preparations containing *C. majus* extract [68,69,70]. *Daphne mezereum* L. has a long history of use in folk medicine and homeopathy. The bark and fruit are used mainly for medicinal purposes [51]. The fruit is used in Greece as a laxative, and the bark is a local irritant [71]. In the traditional medicine of Armenia, the plant is used to counter colitis and some stomach diseases and as an ointment to counter rheumatism and gouty arthritis [51]. *Humulus lupulus* L. has a long history as a medicinal remedy to treat a wide range of complaints [72]. Cones are widely used for medicinal purposes. In Armenia, the plant is used to counter gastritis, diarrhea, pneumonia, hair loss, and furuncles [51,65]․ In France, Germany, and Austria, the cones of the plant are used as a mild sedative, useful for treating insomnia and nervousness [72]. In Poland, the herb is used to treat general weakness, neurasthenia, and diseases of the gastrointestinal tract, while in Bulgaria, it is used to counter sexual excitability, insomnia, lack of appetite, and inflammation of the urinary tract and stones [73]. *Plantago major* L. has a long history of use in folk medicine. The leaves and seeds are mainly used, which are used to counter bronchitis, pulmonary tuberculosis, whooping cough, and a number of diseases of the gastrointestinal tract [50,51]. In France, leaves are used in enterocolitis, tuberculous diarrhoea, and chronic nephritis. In Germany, tea made from leaves is used in upper respiratory tract infections, and a compress made from leaf juice is used for insect bites, furunculosis, and postvaccinal reactions [74]. Red raspberry-*Rubus idaeus* L. is widely known for its edible fruits [75]. The most common herbal remedy in folk medicine is the fruit, partly also the leaves and flowers [76]. In Armenian traditional medicine raspberry fruits are widely used to counter feverish diseases, and the infusion of flowers in the form of compresses are used to counter snake bites [51]. In Austria, a decoction of the fruit and leaves was used as a vitamin drink and also as a cough remedy [77]. In Germany, the leaves are used as a good astringent for diarrhoea and as a gargle for the throat [78]. As an anti-inflammatory, the leaves are used in Bulgaria to counter stomach bleeding, diarrhoea, vomiting, menstrual disorders, and some respiratory diseases [79]. In traditional medicine, the whole plant *Sambucus nigra* L. is used, even the bark and roots, but mainly the flowers and fruits [80]. According to Armenian folk medicine, the flowers of the plant as a diaphoretic and diuretic are useful in uraemia [51,65], while in Czech traditional medicine, the juice of the fruit is used to treat inflammation of the trigeminal and lumbosacral nerves [81]. Some components of *S. nigra* are potentially harmful, e.g., fruits contain a small concentration of ribosome-inactivating protein (RIP), which has minor cytotoxic effects, and a higher concentration in unripe fruits [82,83]. However, careful culinary processing of the fruit before use should denature such proteins [84]. Other cytotoxic effects at very high doses have been suggested, but not at doses that would be recommended for use [85]. In general, the safety concerns of *S. nigra* can be avoided if the plant is properly harvested and the dosage is correct, as reports of adverse effects of *S. nigra* are usually related to the use of a part of the plant not included in the formulation, overdose or consumption of unprepared fruits [84]. *Sanguisorba officinalis* L. has long been used as a traditional medicine [86]. For medicinal purposes, the root and rhizome are mainly used, and sometimes the aerial parts are used [51]. *S. officinalis* is widely used in Asia to treat inflammatory and metabolic diseases, including diarrhoea, chronic intestinal infections, bleeding disorders, and diabetes [87,88]. In Armenian folk medicine, the plant is known as a remedy to stop diarrhoea and intestinal bleeding, regulate menstruation, and normalize blood pressure [51]. In Czech traditional medicine, *S. officinalis* is used as a diuretic, and in Bulgarian traditional medicine, the rhizome is used to treat dysentery and varicose veins of the lower limbs, as well as to stop bleeding [88]․ *Urtica dioica* L․ as a medicinal plant is known to humanity since ancient times [89]. The leaves, flowers, and roots of the plant are used for medicinal purposes. In Armenian traditional medicine, the juice of the plant is mixed with beer and used to relieve coughs and toothache, the root is drunk with water for heartache, and the crushed leaves are applied to the forehead as a compress to stop nosebleeds [51,56]. In Bulgaria, *U․ dioica* is used to counter hair loss, ulcers, diabetes, and chronic bronchitis [90]. In France, the preparation “dioica” obtained from the plant is used to strengthen the hair of the head, as well as in acute and chronic enteritis and diarrhoea of tubercular origin, whereas in Poland and Germany, the plant is well known to counter anaemia, atherosclerosis, rheumatism of joints and muscles, inflammation of the urinary tract, skin, and liver diseases [89].

In the traditional medicine of neighbouring countries, the same plant is used similarly or in different ways. All species of the genus *Allium* are used in the traditional medicine of Armenia [91]. In particular, the aerial parts of *A. ursinum* L. are used as an appetizing, to invigorate the body, and restore strength [49], while the juice of *A. atroviolaceum* Boiss and *A. cepa* L. is used to counter the initial stages of cataract and to improve eyesight [92]. In the traditional medicine of Azerbaijan, *A. ursinum* is used for healing wounds in the form of decoction, gargles, and applications as a disinfectant and wound-healing agent to counter skin diseases, abscesses, and female diseases [93]. Additionally, *A. struzlianum* Ogan. is considered an endemic of Armenia [18]. Another widely used medicinal plant is *Agrimonia eupatoria* L. [94], which is used in Armenia to counter neck tumors, abscesses, inflammatory diseases of the liver and spleen, and abdominal pain [45]․ In Azerbaijan, as well as in Tabriz (Iran), the plant is used to counter gastrointestinal diseases and an aqueous infusion and decoction are used as an astringent to counter diarrhoea, as well as a choleretic to counter inflammation of the gallbladder [95,96]. In Georgia, *A. eupatoria* is used to treat testicular tumours. Additionally, the plant is known as an anthelminthic and for healing wounds, jaundice, and fever [97]. However, care should be taken with the doses used, as consumption of large amounts causes digestive upset and constipation due to the tannin content [98,99].

The fruits and flowers of *Crataegus orientalis* (Mill.) M.Bieb. are used in Armenian folk medicine to counter insomnia, dizziness, heart diseases, and shortness of breath [51], and *C. armena* Pojark. and *C. pallasii* Griseb. are known as remedies for cardiovascular diseases, cancer, and diabetes [30,100]. Whereas in Azerbaijan, *C. curvisepala* is traditionally used in the form of a tea made from unripe fruits and bark of the plant to counter diarrhoea [95], and in Georgia, it is used as a cardiotonic agent in the early stages of hypertension [97]. Another ethnomedicinally important genus is *Centaurea*, represented in Armenia with 46 species, of which six are endemics of Armenia [17]. *C. cyanus* L. and *C. hajastana* Tzvel. (synonym of *Rhaponticoides hajastana* (Tzvelev) M.V.Agab. and Greuter), which is native to the Transcaucasus [30], are used to counter kidney and urinary tract diseases and as an anti-inflammatory to counter eye inflammation [45]. *Lactuca serriola* L., used as a medicinal plant to counter coughs, pneumonia, measles, and rheumatic pains [51], is found in the low and middle mountain belts of Armenia [17]. In Armenia, there are six species of *Lactuca*, including *L. takhtadzhianii* Sosn., a narrow endemic of the *Atropatenic subprovince* of the Armenian–Iranian floristic province [17].

There are more than 800 species of *Rubus* distributed throughout the world [101], but only 13 species of *Rubus* grow in Armenia [11], of which five species are listed in Table S1 as medicinal plants. *R. armeniacus* Focke is very widespread in the Caucasus and used in Armenia to counter leprosy, inflammation of the digestive tract, plague, and burns [45]. In the form of tea, made from the leaves and fruits, *R. caesius* L. and *R. takhtadjanii* Mulk․ are known as a diaphoretic and antipyretic remedy to counter colds [49]. Moreover, *R. takhtadjanii* is an endemic to the Caucasus and has been described from Armenia [11,30]. According to RA Government Decree N 72-N of 2010, the plant species is considered an endangered species and is preserved in the Shikahogh State Reserve.

The fruits of *Sorbus aucuparia* L. are used in Armenian folk medicine as a mild laxative, biliary, and diuretic [51]. *S. hajastana* Gahr., which is an endemic of Armenia, is also used the same way [30]. In Azerbaijan and in Georgia, *S. aucuparia* is known as an antidiarrheal and anti-inflammatory agent [97,102]. Additionally, in Georgia, a decoction of leaves is used to relieve cramps, and the fruit tincture is used to treat heart disease and hypertension and is applied to disinfect wounds [97].

Some of the important medicinal plants used by local people are species of the genus *Thymus*. In Armenia, the aerial parts of *T. kotschianus* Boiss. et Hohen. are used as an appetizer and to help with toothache when eating with food, strengthen eyesight, and regulate breathing [45], while in Azerbaijan they are used to counter cough, bronchitis, angina, flatulence, worms, colds, and flu viruses [103]. The aerial parts of *T. collinus* Bieb. are used similarly to *T. kotschianus* in Armenia [104], whereas in Azerbaijan and in Georgia, an aqueous infusion of the above-ground parts of the species is used internally to treat colds, and as an expectorant [95,105]. In addition, the aerial parts of both species are edible, used as a spice, and for making tea [97]. The young stem is used to make a traditional soup called spas (սպաս), some types of cheese, bread, tea, and vodka in Armenia [106]. The aerial parts of *T. serpyllum* L. and *T. transcaucasicus* Ronn. are used as antiseptic and antispasmodic agents to improve digestion and treat inflammation of the oral cavity [49]. Moreover, *T. transcaucasicus* is endemic to the Caucasus [30] and has the same medicinal usage as in Iğdır (Turkey), Nakhchivan (Azerbaijan), and Tabriz (Iran) [96].

*Vitis sylvestris* C.C.Gmel. is known for medicinal purposes and used in Armenia to counter liver and kidney diseases [46]. However, another species *V. vinifera* L., is widely cultivated, with more than 50 varieties known (Ministry of Economy of RA, 2023 Order No. 20-L). *V. sylvestris* is used as a medicinal plant by the local population also in Azerbaijan. The fruit and fresh juice are used for general weakness and anaemia and to improve appetite. Fresh grape leaves are chewed to treat gum disease, and a water infusion of the leaves is used to rinse the mouth [102].

### 3.5. Endemic Medicinal Plants in the Transcaucasus

**Figure 5:**
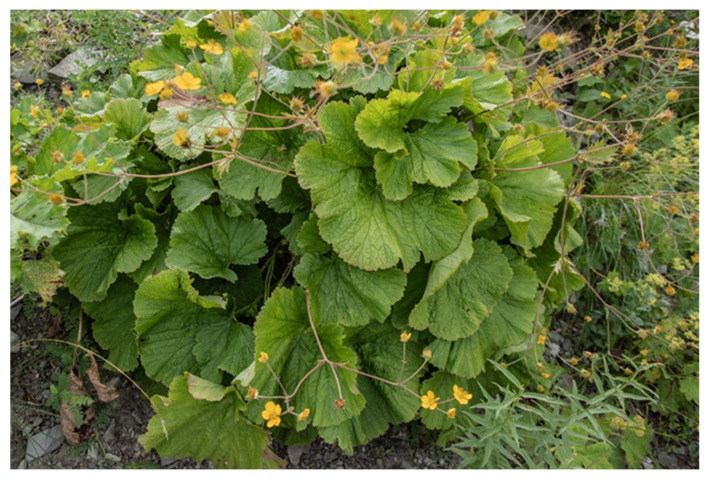
*Woronowia speciosa* (Albov) Juz. (Rosaceae) (photo by Ilya Mikheev).

The Transcaucasian Highlands are located on the border of the Euro–Siberian and Iranian–Turanian biogeographic regions and at the junction of the Caucasian and Iranian-Anatolian biodiversity hotspots. More than 4000 species of vascular plants are found in the Transcaucasian Highlands, of which about 10% are endemics [107], such as *Agasyllis latifolia* (M.Bieb.) Boiss. (Apiaceae) and *Woronowia speciosa* (Albov) Juz. (Rosaceae) (Figure 5), and the endemic genus *Grossheimia* Sosn. and Takht. (Asteraceae) with six species [108].

The flora of Azerbaijan is represented by about 5000 plant species, mostly from the families of Asteraceae, Fabaceae, Poaceae, Lamiaceae, Brassicaceae, and Rosaceae [109]. Of these, nearly 1500 are mentioned in the literature as medicinal plants [110]. The number of plants in Georgia (more than 4500 species) is represented by 900 genera and 140 plant families, and a bunch of plants are used as medicinal [6]. Among these species of medicinal plants, there are endemics of the Caucasus, e.g., *Heracleum asperum* M.Bieb. (Apiaceae), which is widespread in subalpine areas (1800–2500 m) in the Ciscaucasia, on the southern slopes of the Main Range in Eastern Transcaucasia, and Dagestan [105]. A decoction of the roots is mentioned in ethnobotanical studies of Georgia as a remedy for cleansing the body and has been used to treat cancer. In addition, the roots are chewed during toothache [96]. Another endemic species for the Caucasus is *Lilium ledebourii* Boiss. (Liliaceae), which is found in mountain woods [105], and in Azerbaijan, the bulb of the plant is crushed and used externally to treat wounds and burns [111].

*Pyrus caucasica* Fed. (Rosaceae) grows in forests and scrubs from sea level to 1650 m altitude in all regions of the Greater Caucasus and Northern and Central Lesser Caucasus [105]. In Georgia, a decoction of the fruit was used to treat diarrhoea in children, inflammatory diseases of the gastrointestinal tract, and tuberculosis [96], whereas in Azerbaijan, the decoction was used externally to heal wounds [111]. In Armenia, the fruits of the plant are used as food, both fresh and processed (in the form of sauces, jams, pastes, and juices) and dried, e.g., crushed fruits are added to flour for bread [96]. In addition, the fruits are used for the production of fruit wine and vodka [106].

Another endemic of the Caucasus is *Quercus iberica* Stev. (Fagaceae), widespread in the drier areas of eastern Georgia, in the mid-mountain belt of northern Armenia at altitudes up to 1200 m, and also forming forests in the zone between 500–1400 m in Azerbaijan [95]. In Armenia, *Q. iberica* is used to treat diabetes. In addition, the fruit was used medicinally, and it was knocked down and applied to bites of poisonous insects and reptiles to relieve swelling [45]. A decoction of the bark was known in Azerbaijan for its astringent and anti-inflammatory properties [107], and in Georgian folk medicine, a decoction of the bark, leaves, and juice of the trunk of the oak tree is used as an anti-diarrhoeal agent, to treat poisoning, kidney and spleen diseases, as well as, to counter toothache [96]. The subalpine plant *Rhododendron caucasicum* Pall. (Ericaceae) grows at an altitude of 1600 to 3000 m above sea level [105]. In Azerbaijan, the plant is used to counter rheumatism, gout, and fever [107], while in Georgia, a tincture of the leaves is known as a remedy to treat rheumatism, heart and vascular diseases. In addition, tea from the flowers and leaves is used as an anti-inflammatory agent, as well as for the treatment of digestive system diseases [96]. *Symphytum caucasicum* M.Bieb. (Boraginaceae) grows in shrubberies, glades, and the forests of the drying parts of the Caucasus (Ciscaucasia, Dagestan, and Eastern Transcaucasia) [105]. In Armenia, the plant is used as a good remedy to treat inflammation of the sciatic nerve and fractures [112]. However, in Azerbaijan, an aqueous infusion of the root is used to counter diarrhoea, and in Georgia, the leaves and roots are used in ointments and to treat fractures [96].

Recent work has also discussed the potential of antimicrobial metabolites derived from Caucasian medicinal plants as an alternative to traditional antibiotics and that some Georgian medicinal plants are a valuable source of enzymatic, hydrophilic, and hydrophobic antioxidants [113,114].

### 3.6. State-of-the-Art Investigations of Medicinal Plants in Armenia

Many medicinal plant taxa, which have been used in traditional medicine since ancient times, are now beinmg studied in various ways [115]. Researchers are currently discovering novel compounds with medicinal properties in plants, discovering new therapeutic efficacy of herbal preparations, and studying their effects on the human body [116].

Armenian medicinal plants are now actively investigated for various biological activities. Most plants have been studied for their antioxidant and antibacterial activity using acetone, ethanol, or methanol as a solvent for extraction (Table 1). The study of the anti-tumour activity of Armenian medicinal plants is also intensifying [117]. Recently, *Rumex obtusifolius* L. seeds collected from the Tavush region of Armenia (900–1600 m above sea level) showed significant cytotoxic effects to counter human colon adenocarcinoma HT29 and human breast cancer MCF-7 cells [117]. Some herbs, such as *Centaurea hajastana* Tzvel., *Crataegus armena* Pojark., *Heracleum transcaucasicum* Manden., *Hypericum eleonorae* Jelen., *Ribes armenum* Pojark., *Rosa sosnovskyana* Tam., *Rubus takhtadjanii* Mulk., *Sorbus hajastana* Gabrieljan, and *Thymus transcaucasicus* Ronn., which are endemic to the Caucasus, were examined using GC-MS for their bioactive content as well as for antioxidant activity [30,118].

Many species of medicinal plants used in Armenian traditional medicine have also been investigated in clinical studies, mainly on rats and rabbits. Harutyunyan et al. (2014) showed some suppression of contractile (muscle) and noncontractile (intestinal) thermogenesis, as well as a decrease in radiation-convective (vascular) thermal efficiency in rats under the action of an extract from *Cicorium intubus* L. [128]. The anti-inflammatory effect of *Elaeagnus angustifolia* L. seed extracts on xylene-induced ear oedema in rats was studied. The seeds were collected from the Ashtarak municipality during October–November 2004. The data obtained showed a 58% reduction in inflammation compared to diclofenac sodium (71% reduction). The results were also confirmed by histological analysis [129]. The effect of *E. angustifolia* and *Morus alba* L. plant extracts on the microcirculation of the damaged gastric wall was investigated in rats using the model of reserpine-induced ulcer disease. The results showed that *E. angustifolia* extract prevented the reserpine-induced decrease in gastric wall capillary diameter and increased it by 62.75%. It was shown that the number of constricted capillaries decreased to a greater extent when *E. angustifolia* and *M. alba* extracts were used together [130]. Another study investigated the regenerative ability of an ointment derived from the seeds of *E. angustifolia*, which showed that the ointment reduced the burn area on day 8 and day 12 by 29.8% and 71.2%, respectively [131]. An ointment of essential oils extracted from the leaves of *Satureja hortensis* L. was also beneficial for wound regeneration [132]. The positive effect of aqueous extract of *Origanum vulgare* L. on blood glucose level, lipid level, functional liver enzymes (alanine aminotransferase and aspartate aminotransferase) and some biochemical parameters in hyperglycemia induced by immobilization stress in rabbits during 21 days of treatment was shown by Aghajanyan and Tadevosyan (2022) [133]. In another study, an ethanolic extract of *Rumex obtusifolius* L. seeds was shown to have a significant effect on hyperglycemia when administered orally, reducing fasting glucose levels (57.3%, *p* < 0.05) and improving glucose tolerance [134].

Twenty-three percent of all non-prescription drugs in the Armenian pharmaceutical market are herbal medicines [91]. Most of the plant species sold in Armenian markets are used for food (about 150 species). However, about 140 species are sold as medicines or are food species with presumed medicinal value, largely sold as herbal teas [48]. In more detail, more than 100 medicinal products of plant origin are registered in Armenia, of which almost 20 are plant materials, 47 are plant preparations, and about 90 are finished medicinal products of plant origin. In addition, 45 species are sold as ornamental plants, 15 plants are used as a source of wood, and nine species are used as insect repellents [48].

Despite the rich flora and centuries-old experience of folk medicine, the traditional ethnomedical knowledge in Armenia is slowly disappearing due to migration, urbanization, and competition with modern medicine and pharmaceuticals. Many people in Armenia, especially young people, both in the Soviet period and nowadays, tend to avoid traditional medicine and do not believe in its efficacy [50]. However, there are several licensed local producers of herbal medicines in Armenia, e.g., “Antaram” (“Անթառամ”) commercial cooperative, “Khazaros” (“Ղազարոս”) herbs manufacturing enterprise and firm stores, and “Bee” (“Մեղու”) Pharmacy [91]. The species most commonly used as medicines and sold in Yerevan include *Artemisia absinthium* L., *Hypericum perforatum* L., *Mentha longifolia* (L.) L., *Origanum vulgare* L., *Teucrium polium* L., and three species of *Thymus-T. kotschyanus* Boiss. and Hohen., *T. rariflorus* K.Koch, and *T. transcaucasicus* Ronniger. The most common types of remedies are those for the treatment of digestive disorders, the common cold, and other respiratory problems [126]. Hovsepyan et al. presented the attitudes of post-Soviet Armenian society toward traditional knowledge of folk medicine on the example of the Tatev community through interviews with vendors and purchasers at the local market. The study presents 40 names of plant species traditionally used by local people and the sources on the basis of which a particular plant was recognized as medicinal [50].

To be very clear, the traditional use of some species mentioned in this review should be strictly limited and used only under the supervision of an experienced physician, as some of the taxa mentioned are known to have or might have severe side effects. For example, crude extracts of *Teucrium polium* L. showed adverse effects mainly on liver and kidney [135,136,137,138,139]. Some side effects caused by prolonged administration of *Artemisia absinthium* have been reported. For example, neurotoxic effects may occur due to the presence of thujone and its analogues [140]. In addition, long-term use of essential oil derived from *A. absinthium* may cause toxic absinthism, a psychiatric disorder in people with clinical manifestations including seizures, insomnia, and hallucinations [141]. Other side effects may include stomach cramps, dizziness, vomiting, nausea, and anxiety [142].

Eleven species of plants that are used in Armenian traditional medicine (Table S1) are now cultivated: *Anethum graveolens* L. (1 var.), *Allium cepa* L. (4 var.), *Daucus carota* L. (2 var.), *Geranium collinum* Steph. ex Willd. (2 var.), *Juglans regia* L. (1 var.), *Ocimum basilicum* L. (2 var.), *Prunus armeniaca* L. (14 var.), *Punica granatum* L. (2 var.), *Ribes nigrum* L. (1 var.), *Rubus idaeus* L. (1 var.), and *Trifolium pratense* L. (3 var.) (Ministry of Economy of RA, 2023 Order No. 20-L).

## 4. Conclusions

The purpose of the presented review was to provide an insight into the ethnopharmacology of medicinal plants in Armenia. From all available sources, a total of 320 medicinal plant species are listed in this review, belonging to 231 genera and 79 families, with their local names, family names, centuries-long use in traditional medicine by the local people, and parts used. This number of species includes both wild and cultivated plant species. The latter is represented by 11 species widely cultivated in Armenia and having different varieties. It also features medicinal plants that have been known for centuries in other countries as well. The dominant family is the Rosaceae, followed by Asteraceae and Lamiaceae.

Many medicinal plants, which have been used in traditional medicine since ancient times, are now being studied in various ways [116]. Researchers are currently discovering novel compounds with medicinal properties in plants, discovering new therapeutic efficacy of herbal preparations, and studying their effects on the human body [115]. Armenian medicinal plants are now actively investigated for various biological activities. Most plants have been studied for their antioxidant and antibacterial activity using acetone, ethanol, or methanol as solvent for extraction. The study of the anti-tumour activity of Armenian medicinal plants is also intensifying. However, there is precious little data on the phytochemical constituents of these plants. Therefore, it would be interesting to also study the phytochemical constituents of herbs growing in Armenia. In addition, plenty of species still remain unstudied.

Although traditional ethnomedical knowledge in Armenia is gradually disappearing due to migration, urbanization, and competition with modern medicine and pharmaceuticals, there are several licensed local markets in Armenia where about 140 herbal products are sold as medicinal products or are food species with presumed medicinal value, of which almost 20 are plant materials, 47 are plant preparations, and about 90 are finished medicinal products of plant origin. The species most commonly used as medicines and sold in Yerevan include *Artemisia absinthium* L., *Hypericum perforatum* L., *Mentha longifolia* (L.) L., *Origanum vulgare* L., *Teucrium polium* L., and three species of *Thymus-T. kotschyanus* Boiss. and Hohen., *T. rariflorus* K.Koch, and *T. transcaucasicus* Ronniger. However, careful and accurate research is needed to clarify the composition of medicinal plant species biologically active substances, mechanisms of action, and side effects in the treatment of a particular disease. On the other hand, many medicinal species, also endemics, belong to endangered species, are listed in the Red Book of Plants of the RA, and are protected in special reserves, national parks, and state wildlife sanctuaries. Which, in turn, makes it difficult to study them.

## Figures and Tables

**Table 1:** Herbs collected in Armenia investigated for various biological activities.

Plant Species	Family Name	Collection Place	Voucher Specimen Number	Plant Part	Extraction Solvent	Activity/Capability	Method	Reference
*Agrimonia* *eupatoria* L.	Rosaceae	Tavush province (1300–1600 m a.s.l.)	ERCB 13207	The whole plant	Acetone and methanol	Antioxidant	DPPH assay	[119]
						Antimicrobial	Bioautographic assay	[120]
						Hydrogen peroxide (H_2_O_2_) reducing activity	H_2_O_2_ assay	[121]
						Metal chelating activity	-	[121]
					Water, methanol, chloroform, acetone, and hexane	Antimicrobial	Agar well diffusion assay	[121]
*Alchemilla smirnovii* Juz.	Rosaceae	Tavush province (1400–2400 m a.s.l.)		Aerial parts	Ethanol	Cytotoxicity	MTT assay	[100]
*Artemisia dracunculus* L.	Asteraceae	Kotayk province (1600 m a.s.l.)	-	Aerial parts	Ethanol	Radical scavenging activity	DPPH assay	[122]
						Catalase activity	Enzyme assay	[122]
						Peroxisomal acyl-coenzyme A oxidase 1 (ACOX1) activity	UV-spectrophotometric based method	[122]
*Bunias orientalis* L.	Brassicaceae	Tavush province (1400–2400 m a.s.l.)	-	Aerial parts	Ethanol	Cytotoxicity	MTT assay	[100]
*Carum carvi* L.	Apiaceae	Tavush province (1400–2400 m a.s.l.)	-	The whole plant	Ethanol	Cytotoxicity	MTT assay	[100]
*Crataegus pallasii* Griseb.	Rosaceae	Tavush province (1400–2400 m a.s.l.)	-	Fruits, flowers with leaves	Ethanol	Cytotoxicity	MTT assay	[100]
*Gentiana cruciate* L.	Gentianaceae	Tavush province (1400–2400 m a.s.l.)	-	Aerial parts	Ethanol	Cytotoxicity	MTT assay	[100]
*Hypericum alpestre* subsp. *polygonifolium* (Rupr.) Avet. and Takht.	Hypericaceae	Tavush province (1300–1600 m a.s.l.)	ERCB 13206	Aerial parts	Acetone and methanol	Antioxidant	DPPH assay	[93]
						H_2_O_2_ reducing activity	H_2_O_2_ assay	[121]
						Antimicrobial	Bioautographic assay	[120]
					Water, methanol, chloroform, acetone, and hexane	Metal chelating activity	-	[121]
					Methanol	Cytotoxicity	3-(4,5-dimethyltrazol-2-yl)-2,5-diphenyltetrazolium bromide (MTT) assay	[121]
						Catalase activity	Enzyme assay	[121]
Hypericum alpestre subsp. *polygonifolium* (Rupr.) Avet. and Takht.	Hypericaceae	Tavush province (1300–1600 m a.s.l.)	ERCB 13206	Aerial parts	Methanol	Peroxisomal acyl-coenzyme A oxidase 1 (ACOX1) activity	UV-spectrophotometric based method	[121]
						Superoxide Dismutase (SOD) activity	SOD activity assay	[121]
*Inula helenium* L.	Asteraceae	Tavush province (1400–2400 m a.s.l.)	-	Flowers with leaves	Ethanol	Cytotoxicity	MTT assay	[100]
*Lilium armenum* (Miscz. ex Grossh.) Manden.	Liliaceae	Tavush province (1300–1600 m a.s.l.)	ERCB 13209	Stalk with leaf	Water, methanol, chloroform, acetone, and hexane	Antimicrobial	Agar well diffusion assay	[119]
				Bulb	Water, methanol, chloroform, acetone, and hexane	Antimicrobial	Agar well diffusion assay	[119]
*Origanum vulgare* L.	Lamiaceae	Gegharkunik province (1930 m a.s.l.)	ERE191395	Aerial parts	Ethanol	Chelating	-	[123]
						Antioxidant	TBARS assay	[123]
						Tyrosinase inhibitory	Tyrosinase Inhibition	[123]
						Antimicrobial	Broth dilution	[123]
						Cytotoxicity	MTT assay	[124]
*Ribes nigrum* L.	Grossulariaceae	Lori province (1600–1650 m a.s.l.)	-	Leaf	Ethanol	Antimicrobial	Disk-diffusion method	[125]
						Radical scavenging capacity	DPPH assay	[125]
						Cytotoxicity	MTT assay	[126]
						Genotoxic effects	Comet assay	[126]
*Ribes rubrum* L.	Grossulariaceae	Lori province (1600–1650 m a.s.l.)	-	Leaf	Ethanol	Antimicrobial	Disk-diffusion method	[125]
						Radical scavenging capacity	DPPH assay	[125]
*Rumex obtusifolius* L.	Polygonaceae	Tavush province (1300–1600 m a.s.l.)	ERCB 13208	Seed	Acetone and methanol	Antioxidant	DPPH assay	[93]
						Hydrogen peroxide (H_2_O_2_) reducing activity	H_2_O_2_ assay	[121]
					Water, methanol, chloroform, acetone and hexane	Metal chelating activity	-	[121]
				Inflorescence	Water, methanol, chloroform, acetone and hexane	Antimicrobial	Agar well diffusion assay	[119]
*Rumex obtusifolius* L.	Polygonaceae	Tavush province (1300–1600 m a.s.l.)	ERCB 13208	Leaf	Water, methanol, chloroform, acetone and hexane	Antimicrobial	Agar well diffusion assay	[119]
*Rumex obtusifolius* L.	Polygonaceae	Tavush province (1300–1600 m a.s.l.)	ERCB 13208	Root	Water, methanol, chloroform, acetone, and hexane	Antimicrobial	Agar well diffusion assay	[119]
					Acetone and methanol	Antimicrobial	Bioautographic assay	[120]
*Sanguisorba officinalis* L.	Rosaceae	Tavush province (1300–1600 m a.s.l.)	ERCB 13205	Aerial parts	Acetone and methanol	Antioxidant	DPPH assay	[93]
						Antimicrobial	Bioautographic assay	[120]
						Hydrogen peroxide (H_2_O_2_) reducing activity	H_2_O_2_ assay	[121]
					Water, methanol, chloroform, acetone, and hexane	Metal chelating activity	-	[121]
				Root	Water, methanol, chloroform, acetone, and hexane	Antimicrobial	Agar well diffusion assay	[119]
*Thymus serpyllum* L.	Lamiaceae	Goris region (650–2400 m a.s.l.)	-	Leaf and flower	Acetone, benzol, chloroform, diethyl ether, ethyl acetate, methanol	Antioxidant	Kinetics	[127]

## Supplementary Materials

The following supporting information can be downloaded at: https://www.mdpi.com/article/10.3390/plants13233411/s1, Table S1: Plant taxa referred to as Armenian traditional medicine in various sources. Table S2: Common names of plant species used in Armenian traditional medicine. References [143,144,145,146,147,148,149] are cited in the Supplementary Materials.

## Abbreviations

ACOX1	Peroxisomal acyl-coenzyme A oxidase 1
AD	Anno Domini
a.s.l.	Above Sea Level
BC	Before Christ
DPPH	2,2-diphenyl-1-picrylhydrazyl
H2O2	Hydrogen peroxide
MTT	3-(4,5-dimethyltrazol-2-yl)-2,5-diphenyltetrazolium bromide)
NAS	National Academy of Science
RA	Republic of Armenia
RIP	Ribosome-inactivating protein
SSS	Student Scientific Society
TBARS	Thiobarbituric Acid Reactive Species Assay
YSU	Yerevan State University
