# Supplementary material for: Traditionally Used Medicinal Plants of Armenia
Source: Plants (Basel). 2024 Dec 4;13(23):3411. doi: 10.3390/plants13233411 (PMC11644142; doi:10.3390/plants13233411)
Supplement: Supplementary file 1 [file plants-13-03411-s001.zip › plants-3322294-supplementary.pdf]

plants

Review

Supplementary material

Traditionally used medicinal plants of Armenia

Arpine Ayvazyan[1] and Christian Zidorn[1,2,*]

[1] Pharmazeutisches Institut, Abteilung Pharmazeutische Biologie, Christian-Albrechts-Universität zu Kiel, Gutenbergstraße 76, 24118 Kiel, Germany; ayvazyanarpine95@gmail.com (A. Ayvazyan), czidorn@pharmazie.uni-kiel.de (C. Zidorn)
[2] Division of Pharmaceutical Biotechnology, Department of Pharmaceutical Biology and Biotechnology, Wroclaw Medical University, Borowska 211, 50-556 Wrocław, Poland
* Correspondence: czidorn@pharmazie.uni-kiel.de; Tel.: +49 431 880-1139

Citation: To be added by editorial staff during production.

Academic Editor: Firstname Lastname

Received: date
Revised: date
Accepted: date
Published: date

Copyright: © 2024 by the authors. Submitted for possible open access publication under the terms and conditions of the Creative Commons Attribution (CC BY) license (https://creativecommons.org/licenses/by/4.0/).

a References are in the manuscript

plants MDPI

Table S1. Plant taxa referred to as Armenian traditional medicine in various sources.

**Botanical name**	**Local name** (in Latin/in Armenian)	**Family name**	**Mainly used part**	**Traditional use**	**References**[a]
Achillea millefolium L.	Hazaraterevuk sovorakan, byuraterevuk/Հազարատերևուկ սովորական, բյուրատերևուկ	Asteraceae	Aerial parts	Infusion is used against diseases of the upper respiratory tract, dysentery and as hemostatic during uterine bleeding.	[51,56]
Acorus calamus L.	Baxshtak/Բաղշտակ	Acoraceae	Rhizome	Used as an appetizing, analgesic, astringent and disinfectant.	[45,51]
Adonis aestivalis L.	Kuzhkotruk amarrayin/Կուժկոտրուկ ամառային	Ranunculaceae	Stem, leaves, and flowers	Used as diuretic, analgesic and hemostatic. Mainly is used against cardiovascular insufficiency.	[65,92]
Adonis vernalis L.	Kuzhkotruk garnanayin/Կուժկոտրուկ գարնանային	Ranunculaceae	Stem, leaves, and flowers	The water decoction is used as a regulator of the working of the heart.	[45]
Aesculus hippocastanum L.	Dziakask, dziashaganakeni/Ձիակասկ, ձիաշագանակենի	Sapindaceae	Trunk and branches bark, leaves, and flowers	Decoction of the flowers, leaves and bark is used against venous and rheumatic diseases, and arthritis.	[45,65]
Agrimonia eupatoria L.	Yeresnak deghatu, yerizhnak/Երեսնակ դեղատու, երիժնակ	Rosaceae	Aerial parts	Used against neck tumors, abscesses, inflammatory diseases of the liver and spleen, stomach-ache.	[45,143]

a References are in the manuscript

Table S1 (*continued*)

**Botanical name**	**Local name** (in Latin/in Armenian)	**Family name**	**Mainly used part**	**Traditional use**	**References**[a]
Agropyron repens (L.) P.Beauv.	Sez soghatsvogh/Սեզ սողացող	Poaceae	Rhizome	The decoction of the rhizome is used against diseases of the urinary and biliary tracts, the gastrointestinal tract, and the skin.	[112]
Alchemilla smirnovii Juz.[b]	Gaylatat Smirnovi/Գայլաթաթ Սմիրնովի	Rosaceae	Aerial parts	Used against purulent wounds, and eyelid inflammations.	[56,106]
Alhagi persarum Boiss. et Buhse	Ughtapush parskakan/Ուղտափուշ պարսկական	Fabaceae	Aerial parts and roots	Used as a choleretic, and laxative.	[45,65]
Alhagi pseudalhagi (Boiss. and Buhse) Takht.[b]	Ughtapush sovorakan, arak/Ուղտափուշ սովորական, արաք	Fabaceae	Aerial parts and roots	Used as a choleretic, and laxative.	[45,65]
Alkanna orientalis (L.) Boiss.	Havajiva/Հավաջիվա	Boraginaceae	Roots	A paste made by boiling the root with oil is used against wounds and ulcers.	[143,144]
Alliaria petiolata (Bieb.) Cavara et Grande	Skhtorabuys deghatu/Սխտորաբույս դեղատու	Brassicaceae	Leaves and seeds	Fresh leaves and decoction are used against wounds, ulcers, and skin diseases.	[45,63]

[a] References are in the manuscript; [b] endemic to the Caucasus.

Table S1 (*continued*)

**Botanical name**	**Local name** (in Latin/in Armenian)	**Family name**	**Mainly used part**	**Traditional use**	**References**[a]
Allium ursinum L.	Sokh arji, arjaskhtor/Սոխ արջի, արջասխտոր	Amaryllidaceae	Aerial parts	Used as an appetizing, to invigorate the body, and restore strength.	[144,145]
Allium atroviolaceum Boiss	Sokh mug manushakaguyn, karm-rasokhuk/Սոխ մուգ մանուշակագույն, կարմրասոխուկ	Amaryllidaceae	The whole plant, mainly bulbs	The juice used in the initial stages of cataracts. Onions improve eyesight.	[45,65]
Allium cepa L.	Sokh sovorakan, glukh sokh/Սոխ սովորական, գլուխ սոխ	Amaryllidaceae	The whole plant, mainly bulbs	The juice used in the initial stages of cataracts. Onions improve eyesight.	[56,92]
Althaea officinalis L.	Tught deghatu, aghtek/Տուղտ դեղատու, աղթեկ	Malvaceae	Mainly roots	The decoction of the roots was used against sore throats. The same decoction, with wine or milk, was used against urinary stones.	[63,46]
Althaea armeniaca Ten.	Tught haykakan/Տուղտ հայկական	Malvaceae	Mainly roots	The decoction of the roots was used against sore throats. The same decoction, with wine or milk, was used against urinary stones.	[65,106]
Amaranthus retroflexus L.	Zimel, aqlorabbuk/Ձիմել, աքլորաբբուկ	Amaranthaceae	Flowers and roots	The aqueous decoction is used against diseases of the gastrointestinal tract. It is used as a hemostatic, and laxative.	[45,143]
Amygdalus fenzliana (Fritsch) Lipsky [b]	Nsheni Fehntsil/Նշենի Ֆեհնցիլ	Rosaceae	Seeds	Almond oil is used against diabetes, anaemia, insomnia, and migraine.	[45,56]

[a] References are in the manuscript; [b] endemic to the Caucasus.

Table S1 (*continued*)

**Botanical name**	**Local name** (in Latin/in Armenian)	**Family name**	**Mainly used part**	**Traditional use**	**References**[a]
Anagallis arvensis L.	Mknakanj dashtayin/Մկնականջ դաշտային	Primulaceae	Aerial parts	Used as a choleretic, anti-inflammatory, sedative, and analgesic. The sap of the plant is used against wounds, and various tumours, as well as eye diseases (especially in the early stages of cataracts).	[106,63]
Anchusa azurea Mill.	Shikkhar, hortalezu italakan/Շիկխար, հորթալեզու իտալական	Boraginaceae	Leaves, stems and roots	A decoction or tincture was used as an emollient against various swellings and pneumonia.	[65,92]
Anethum graveolens L.	Teraghotik/Տերաղոթիկ	Apiaceae	Leaves and seeds	Decoction of leaves and seeds is used as a diuretic. Also, it is used in the early stages of hypertension.	[145,146]
Anthriscus cerefolium (L.) Hoffm.	Kerbeluk momaterev/Կերբելուկ մոմատերև	Apiaceae	Aerial parts, sap, flowers and fruits	Tincture of the leaves is used against bronchitis and pneumonia.	[65,144]
Apium graveolens L.	Lakhur, kyarauz/Լախուր, քյարաուզ	Apiaceae	Fruits and roots	The juice is used as a diuretic, appetizing, analgesic, and to improve the gastrointestinal tract.	[45,63]
Artemisia abro-tanum L.	Oshindr buzhich /Օշինդր բուժիչ	Asteraceae	Fresh leaves and rhizomes	Decoction of the leaves is used as an appetizing, diuretic and laxative, and used against anaemia, fever, rheuma-tism, haemorrhoids, and tuberculosis.	[45,143]

[a] References are in the manuscript; [b] endemic to the Caucasus.

Table S1 (*continued*)

**Botanical name**	**Local name** (in Latin/in Armenian)	**Family name**	**Mainly used part**	**Traditional use**	**References**[a]
Artemisia absinthium L.	Oshindr darry/Օշինդր դառը	Asteraceae	Leaves and flower bearer	Used against headaches, eye pain, and bone pain. The herb mixed with honey helps with jaundice, earache, helps to avoid getting drunk, improves hearing, opens the urinary tract.	[56,106]
Artemisia dracunculus L.	Tarkhun/Թարխուն	Asteraceae	Aerial parts	Used as an appetizing, and to regulate metabolism.	[46,146]
Arum maculatum L.	Nvik btsavor/Նվիկ բծավոր	Araceae	Fresh tuberous rhizomes	A warm infusion is used against rheumatic arthritis, and a powder from the dried rhizome used against old wounds.	[45,63]
Arum orientale M.Bieb.	Nvik arevelyan/Նվիկ արևելյան	Araceae	Rhizomes, leaves, and fruits	Used against diarrhea, coughs. Mixed with honey is used as a smear, useful against scabies and freckles.	[51,56]
Asparagus officinalis L.	Tsnepak deghatu, marazho/Ծնեփակ դեղատու, մարաժո	Asparagaceae	Roots and shoots	Herbal preparations are used to open clogged veins, against liver and kidney diseases, lumbar pain, and toothache.	[51,63]
Asparagus verticillatus L.	Tsnepak oghakadzev/Ծնեփակ օղակաձև	Asparagaceae	Roots and shoots	Herbal preparations are used to open clogged veins, against liver and kidney diseases, lumbar pain, and toothache.	[51,63]

[a] References are in the manuscript; [b] endemic to the Caucasus.

Table S1 (*continued*)

**Botanical name**	**Local name** (in Latin/in Armenian)	**Family name**	**Mainly used part**	**Traditional use**	**References**[a]
Asperula odorata L.	Getnastgh, asprik/Գետնաստղ, ասպրիկ	Rubiaceae	Aerial parts	Used as an antispasmodic and sedative against insomnia, hypertension, kidney, liver, and gallbladder problems.	[21,65]
Astragalus microcephalus Willd.	Gaz manraglkhik/Գազ մանրագլխիկ	Fabaceae	Aerial parts	Long-term use of water infusion relieves heart attacks, regulates blood pressure, and heart rate.	[51,145]
Astragalus strictifolius Boiss.	Gaz ughghaterev/Գազ ուղղատերև	Fabaceae	Aerial parts	Long-term use of water infusion relieves heart attacks, regulates blood pressure, and heart rate.	[56,106]
Barbarea vulgaris W.T.Aiton	Ktsmndzuk agheghnadzev, hladzuk/Կծմնծուկ աղեղնաձև, հլածուկ	Brassicaceae	Aerial parts	The decoction is used against paralysis, avitaminosis, and impotence.	[45,65]
Berberis iberica Steven & Fisch. ex DC. [b]	Tsoreni vratsakan/Ծորենի վրացական	Berberidaceae	Roots and flowers	The root extract is used against intestinal obstruction, and crushed roots and fruits are used against tuberculosis.	[56,144]
Berberis orientalis C.Schneid. [b]	Tsoreni arevelyan/Ծորենի արևելյան	Berberidaceae	Roots and flowers	The root extract is used against intestinal obstruction, and crushed roots and fruits are used against tuberculosis.	[143,144]
Berberis vulgaris L.	Tsoreni sovorakan/Ծորենի սովորական	Berberidaceae	Roots and flowers	The root extract is used against intestinal obstruction, and crushed roots and fruits are used against tuberculosis.	[51,146]

[a] References are in the manuscript; [b] endemic to the Caucasus.

Table S1 (*continued*)

**Botanical name**	**Local name** (in Latin/in Armenian)	**Family name**	**Mainly used part**	**Traditional use**	**References**[a]
Betonica officinalis L.	Ttvich deghatu/Թթվիճ դեղատու	Lamiaceae	Aerial parts with flower-heads	Decoction is used against liver, stomach, kidney, gallbladder and biliary tract diseases. Fresh leaves are used against wounds and burns.	[51,56]
Betula litwinowii (Doluch.) Ashburner & McAll.	Kechi Litvinovi/Կեչի Լիտվինովի	Betulaceae	Buds and young leaves	Birch preparations are used against stone formation in kidney and bladder. The leaves and kidneys are used to brew a tea, which is used as a diuretic and anti-inflammatory.	[63,147]
Betula pendula Roth	Kechi lalkan, tghteni/Կեչի լալկան, թղթենի	Betulaceae	Buds and young leaves	Birch preparations are used against stone formation in kidney and bladder. The leaves and kidneys are used to brew a tea, which is used as a diuretic and anti-inflammatory.	[63,147]
Betula pubescens Ehrh.	Kechi tavot/Կեչի թավոտ	Betulaceae	Buds and young leaves	Birch preparations are used against stone formation in kidney and bladder. The leaves and kidneys are used to brew a tea, which is used as a diuretic and anti-inflammatory.	[45,56]
Bifora radians M.Bieb.	Gndzik charragaytavor/Գնձիկ ճառագայթավոր	Apiaceae	Flowers, fruits, and aerial parts	Tincture of the leaves is used against hepatitis and jaundice. Fresh leaves are used externally against boils and skin diseases.	[56,147]
Brassica juncea (L.) Czern.	Mananekh sareptakan/Մանանեխ սարեպտական	Brassicaceae	Seeds	Preparations of the plant are used against various skin diseases.	[65,92]

[a] References are in the manuscript; [b] endemic to the Caucasus.

Table S1 (*continued*)

**Botanical name**	**Local name** (in Latin/in Armenian)	**Family name**	**Mainly used part**	**Traditional use**	**References**[a]
Bryonia alba L.	Loshtak spitak, marmnatak/Լոշտակ սպիտակ, մարմնատակ	Cucurbitaceae	Mainly roots	Used as an analgesic, hemostatic, purgative, and diuretic.	[51]
Bryonia dioica (Jacq.) Tutin	Yerktun loshtak/Երկտուն լոշտակ	Cucurbitaceae	Roots	Used as an analgesic, hemostatic, purgative, and diuretic.	[45,65]
Bunias orientalis L.	Ktsvuk/Կծվուկ	Brassicaceae	Aerial part	Used against wounds, hypertension, infertility, lung oedema, diarrhea, and tumours.	[63,100]
Butomus umbellatus L.	Karapatsaghik hovanotsayin/Կարապածաղիկ հովանոցային	Butomaceae	Rhizomes, leaves, and seeds	Decoction of the rhizomes is used against inflammation of the kidneys, respiratory tract, and liver. A decoction of the seeds used against swelling.	[144,145]
Caltha palustris L.	Tsing chahchayin, aytsarratunk/Ցինգ ճահճային, այծառատունկ	Ranunculaceae	Leaves and flower-buds	The sap obtained from fresh leaves and flowers-buds is used as an emollient. The leaves are also used in the form of decoction as an anti-inflammatory, analgesic, and also as a diuretic. The fresh aerial parts are used externally against burns, inflamed skin, and injuries.	[144,146]
Caltha polypetala Hochst.	Tsungi bazmatert/Ցունգի բազմաթերթ	Ranunculaceae	Leaves and flower-buds	The sap obtained from fresh leaves and flowers-buds is used as an emollient. The leaves are also used in the form of decoction as an anti-inflammatory, analgesic, and also as a diuretic. The fresh aerial parts are used externally against burns, inflamed skin, and injuries.	[65,144]

[a] References are in the manuscript; [b] endemic to the Caucasus.

Table S1 (*continued*)

**Botanical name**	**Local name** (in Latin/in Armenian)	**Family name**	**Mainly used part**	**Traditional use**	**References**[a]
Calystegia sepium (L.) R.Br.	Tipatatuk tsankapatayin/Տիպատատուկ ցանկապատային	Convolvulaceae	Roots and aerial parts	Used as a hemostatic, anthelmintic, analgesic, and laxative. As an external application (compresses, wetting), decoction of the ground part is used against injuries, wounds, and ulcers.	[51,65]
Capparis spinosa L.	Kapar pshot, kapareni/Կապար փշոտ, կապարենի	Capparaceae	Roots and aerial parts	The sap obtained from the fresh leaves is used against ear infections, and the roots are used against spleen diseases.	[145,147]
Capsella bursa-pastoris Medik.	Hovvamaghakh/Հովվամաղախ	Brassicaceae	Aerial parts	The aqueous decoction is used as a hemostatic during kidney, lung, and uterine bleeding.	[106,63]
Carex brevicollis DC.	Boshkh karchakit/Բոշխ կարճաքիթ	Cyperaceae	Rhizomes and leaves	Decoction of the rhizomes and leaves is used as an expectorant against rheumatism, furunculosis, gout, pneumonia, and female diseases.	[20,145]
Carum carvi L.	Haychaman, chapurr/Հայչաման, ճապուռ	Apiaceae	Fruits	Decoction of the fruit is used against functional disorders of the nervous system of the gastrointestinal tract, diseases of the liver and gallbladder.	[65,106]
Castanea sativa Mill.	Shaganak tsanovi, shahbalut/Շագանակ ցանովի, շահբալուտ	Fagaceae	Leaves, flowers, rip fruits, and trunk barks	Tea and decoction from the leaves are used against severe coughs, whooping cough, asthma, and other respiratory ailments.	[46,143]

[a] References are in the manuscript; [b] endemic to the Caucasus.

Table S1 (*continued*)

**Botanical name**	**Local name** (in Latin/in Armenian)	**Family name**	**Mainly used part**	**Traditional use**	**References**[a]
Celtis caucasica Willd. [b]	Prrshni kovkasyan/Փռշնի կովկասյան	Cannabaceae	Leaves, seeds, and trunk barks	The seeds are used as a diaphoretic, and the leaves as an antiseptic.	[51,146]
Celtis glabrata Stev. ex Planch.	Merkaterev prrshni/Մերկատերև փռշնի	Cannabaceae	Leaves, seeds, and trunk barks	The seeds are used as a diaphoretic, and the leaves as an antiseptic.	[51,146]
Centaurea cyanus L.	Kaptatsaghik/Կապտածաղիկ	Asteraceae	Flowers	Decoction of the flowers is used against kidney and urinary tract diseases, and as an anti-inflammatory against eye inflammation.	[45,146]
Centaurea hajastana Tzvel. [b]	Kaptatsaghik haykakan/Կապտածաղիկ հայկական	Asteraceae	Flowers	Decoction of the flowers is used against kidney and urinary tract diseases, and as an anti-inflammatory against eye inflammation.	[30,56]
Centaurium erythraea Rafn	Darrnaleghi eritrea/Դառնալեղի էրիտրեա	Gentianaceae	Aerial parts	Used against vomiting, gastritis, diabetes, jaundice and kidney disease.	[45,100]
Cerasus avium (L.) Moench	Kerraseni antarrayin, tstabali/Կեռասենի անտառային, ծտաբալի	Rosaceae	Leaves, fruits, resin, and root bark	The resin used against coughs, lung ulcers, bronchial asthma and colds, while decoction of the roots is used against stomach ulcers.	[56,148]
Chelidonium majus L.	Kanteghakhot mets, odzi aryun/Կանթեղախոտ մեծ, օձի արյուն	Papaveraceae	Aerial parts	Used against arthritis, headaches, especially migraines.	[51,65]

[a] References are in the manuscript; [b] endemic to the Caucasus.

Table S1 (*continued*)

**Botanical name**	**Local name** (in Latin/in Armenian)	**Family name**	**Mainly used part**	**Traditional use**	**References**[a]
Chenopodium album L.	Teluk spitak, sagakhot/Թելուկ սպիտակ, սագախոտ	Amaranthaceae	Aerial parts, leaves and seeds	Fresh sap is used against abdominal pain, headaches and diarrhoea.	[46,148]
Chondrilla juncea L.	Tsamanik/Ծամանիկ	Asteraceae	Roots, aerial parts and flowers	Decoction of the leaves is used against menstrual disorders, decoction of the leaves and roots against diarrhoea. It is also used as an antidote against venomous snake bites.	[56,144]
Cichorium intybus L.	Yegherdak, aytskhot/Եղերդակ, այծխոտ	Asteraceae	The whole plant, manly roots	The tar obtained from the plant is used against various skin diseases (old wounds, ulcers, abscesses).	[51,63]
Cornus mas L.	Hon sovorakan/Հոն սովորական	Cornaceae	Leaves, barks, roots and fruits	The fruit and leaves are used against metabolic disorders, gout and skin diseases, while decoction of the root and bark is used against rheumatism and malaria.	[144,146]
Coronilla varia L.	Kararrvuyt yerpnerang, khachatur tsaghik/Քարառվույտ երփներանգ, խաչատուր ծաղիկ	Fabaceae	Aerial parts and seeds	In the form of a tea or tincture, the crushed part is used as a diuretic and heart-regulating agent.	[45,143]
Corylus avellana L.	Tkhleni/Տխլենի	Betulaceae	Fruits, leaves and branch bark	Decoction of the leaves is used against liver and gallbladder diseases.	[144,145]
Cotinus coggygria Scop.	Narnjapayt/Նարնջափայտ	Anacardiaceae	Green leaves	Decoction of the leaves is used against hemorrhoids, inflammatory wounds, and some skin diseases.	[46,146]

[a] References are in the manuscript; [b] endemic to the Caucasus.

Table S1 (*continued*)

**Botanical name**	**Local name** (in Latin/in Armenian)	**Family name**	**Mainly used part**	**Traditional use**	**References**[a]
Cotoneaster integerrimus Medik.	Chmeni amboghjayezr/Չմենի ամբողջաեզր	Rosaceae	Rhizomes, leaves, and fruits	Decoction of the fruit is used against dysentery and flatulence. It is also used as an astringent against gastric ulcers and enteritis.	[106,143]
Cotoneaster melanocarpus Fisch. ex Loudo	Chmeni sevaptugh/Չմենի սևապտուղ	Rosaceae	Rhizomes, leaves, and fruits	Decoction of the fruit is used against dysentery and flatulence. It is also used as an astringent against gastric ulcers and enteritis.	[65,92]
Crataegus armena Pojark. [b]	Haykakan szni/Հայկական սզնի	Rosaceae	Fruits	Used against cardiovascular diseases, cancer, diabetes.	[30,46]
Crataegus orientalis (Mill.) M.Bieb.	Szni arevelyan, krkteni/Սզնի արևելյան, կրկտենի	Rosaceae	Flowers and ripe fruits	The fruits powder or crushed or in the form of tea, and the flowers in the form of tea are used against insomnia, dizziness, heart diseases, shortness of breath.	[51,143]
Crataegus pallasii Griseb.	Alocheni Pallas/Ալոճենի Պալլաս	Rosaceae	Fruits, flowers with leaves	Used against cardiovascular diseases, cancer, diabetes.	[145,100]
Cuscuta epithymum L.	Gaghdz sovorakan/Գաղձ սովորական	Convolvulaceae	The whole plant	Used as a choleretic and used against neuropsychiatric diseases. Used also against malaria, fever, gallbladder inflammation, and spleen diseases.	[51,65]
Cuscuta europaea L.	Gaghdz yevropakan/Գաղձ եվրոպական	Convolvulaceae	The whole plant	Used against seizures, worm infestations and white fever, as well as to remove moles and freckles.	[45,63,65]

[a] References are in the manuscript; [b] endemic to the Caucasus.

Table S1 (*continued*)

**Botanical name**	**Local name** (in Latin/in Armenian)	**Family name**	**Mainly used part**	**Traditional use**	**References**[a]
Cuscuta monogyna Vahl	Gaghdz miasrrnakani/Գաղձ միասռնականի	Convolvulaceae	The whole plant	Used as a diuretic, analgesic, and laxative.	[45,65]
Daphne mezereum L.	Gaylahat mahatsu/Գայլահատ մահացու	Thymelaeaceae	Barks and fruits	Used against colitis and some stomach diseases. As an ointment it is used against rheumatism and gout.	[51,143]
Datisca cannabina L.	Kanep vayri, jrkanep/Կանեփ վայրի, ջրկանեփ	Datiscaceae	Aerial parts and seeds	Decoction of the seeds is used as a laxative and vomiting agent.	[46,147]
Datura stramonium L.	Arjynkuyz garshahot, satani tuz/Արջընկույզ գարշահոտ, սատանի թուզ	Solanaceae	Leaves	A small amount of decoction of leaves and seeds is used as a sedative against colon diseases, rheumatism, to relieve cramps, and whooping cough.	[144,147]
Daucus carota L.	Gazar/Գազար	Apiaceae	Seeds, roots, and leaves	Used against inflammatory diseases of the stomach, liver, spleen, digestive disorders.	[46,65]
Dictamnus caucasicus Fisch. ex Grossh. [b]	Khndan, voghkuzak/Խնդան, ողկուզակ	Rutaceae	The whole plant	The decoction is used as a diaphoretic against kidney stones, gastrointestinal diseases, rheumatism and some skin diseases. The decoction of the roots is used as an analgesic.	[144,146]
Digitalis ferruginea L. [b]	Matnotsuk zhangot, puch-pucha/Մատնոցուկ ժանգոտ, պուճպուճա	Plantaginaceae	Leaves	Used against heart diseases.	[45,65]

[a] References are in the manuscript; [b] endemic to the Caucasus.

Table S1 (*continued*)

**Botanical name**	**Local name** (in Latin/in Armenian)	**Family name**	**Mainly used part**	**Traditional use**	**References**[a]
Diphelypaea coccinea (M.Bieb.) Nicolson	Yeghbayraryun karmir/Եղբայրարյուն կարմիր	Orobanchaceae	The whole plant	Used against gangrene, various tumors, ulcers, uterine, and gastrointestinal diseases.	[46,63]
Dipsacus strigosus Willd. ex Roem. & Schult.	Akkan, hovvi sanr/Ակքան, հովվի սանր	Caprifoliaceae	Leaves, stems and flowers or flower-heads	The decoction is used as an anti-inflammatory and diuretic, and to improve the cardiovascular, respiratory, and circulatory systems.	[51,56]
Dracocephalum moldavica L.	Vishapaglux moldavakan/Վիշապագլուխ մոլդավական	Lamiaceae	Aerial parts and flowers	The decoction is used as an anti-inflammatory, wound-healing agent, and analgesic against headaches and as a remedy against bloating.	[51,65,146]
Echinops sphaerocephalus L.	Vozneni kloraglukh/Ոզնենի կլորագլուխ	Asteraceae	Seeds	Decoction of the seeds and congee of the fruits are used against headaches, epilepsy, and sclerosis. Fatty oils are used externally against various skin diseases.	[46,56]
Echium vulgare Brot.	Izhakhot sovorakan/Իժախոտ սովորական	Boraginaceae	Stems, flowers, and leaves	The decoction is used as an expectorant against coughs, laryngitis and whooping cough, whereas decoction of the roots is used against rheumatism and joint pains.	[51,148]
Elaeagnus angustifolia L.	Pshateni neghaterev/Փշատենի նեղատերև	Elaeagnaceae	Seeds, flowers and leaves	Flowers and seeds were used against oedema, scurvy, as an anthelmintic, against colitis, bronchitis, heart disease. Leaves are used against rheumatism and gout pains, as well as against wounds.	[46,63]

[a] References are in the manuscript; [b] endemic to the Caucasus.

Table S1 (*continued*)

**Botanical name**	**Local name** (in Latin/in Armenian)	**Family name**	**Mainly used part**	**Traditional use**	**References**[a]
Elytrigia repens (L.) Nevski.	Sez soghatsvogh/Սեզ սողացող	Poaceae	Rhizomes	Decoction of the rhizome is used against metabolic disorders, skin diseases and furunculosis. Mainly in the form of a tea, as an expectorant used in the treatment of inflammation of the respiratory tract and biliary tract.	[63,92]
Ephedra distachya L.	Sari chamich sovorakan/Սարի չամիչ սովորական	Ephedraceae	Aerial parts	A small amount of decoction is used against rheumatism, malaria, and respiratory diseases (e.g., asthma, whooping cough).	[56,144]
Equisetum arvense L.	Dziadzet dashtayin, dziagi/Ձիաձետ դաշտային, ձիագի	Equisetaceae	Aerial parts	Used against bleeding, haemorrhoids, and hard-to-heal wounds.	[45,63,146]
Eremurus spectabilis M.Bieb. [b]	Shresh ushagrav, paylktuk/Շրեշ ուշագրավ, փայլկտուկ	Asphodelaceae	Roots	Powder obtained from the roots is used against abscesses and some kind of tumours.	[46,65]
Eruca vesicaria (L.) Cav.	Charchruk, asoreak/Ճարճրուկ, ասորեակ	Brassicaceae	Aerial parts, flowers, and seeds	A decoction of the seeds is used to remove pustules and boils, in addition, to regulate the digestive system.	[46,148]
Eryngium campestre L.	Yernjak/Երնջակ	Apiaceae	Roots	Fresh root sap or hot tincture of crushed roots used as diuretic, antispasmodic and analgesic, and enhances the menstrual cycle. It is also used against purulent blisters and wounds.	[49,148]

[a] References are in the manuscript; [b] endemic to the Caucasus.

Table S1 (*continued*)

**Botanical name**	**Local name** (in Latin/in Armenian)	**Family name**	**Mainly used part**	**Traditional use**	**References**[a]
Euphrasia hirtella Jord. ex Reut.	Aknakhot mazot/Ակնախոտ մազոտ	Orobanchaceae	Aerial parts and flowers	Used as an anti-inflammatory and general tonic against strokes, in the treatment of eye diseases and digestive disorders.	[51,145]
Filipendula hexapetala Gilib.	Prpruk vetstertikavor/Փրփրուկ վեցթերթիկավոր	Rosaceae	Aerial parts and rhizomes with roots	Decoction is used against wounds for gout, rheumatism and skin diseases. Decoction of roots and rhizomes is used against leukaemia, diarrhoea, and some female illnesses.	[148]
Filipendula ulmaria (L.) Maxim.	Prpruk teghanman/Փրփրուկ թեղանման	Rosaceae	Aerial parts or flower-heads	Decoction of the aerial parts and flowers is used against rheumatic pains, gout, and arthritis.	[51,146]
Foeniculum vulgare Mill.	Rrazian, horrom samit/Ռազիան, հոռոմ սամիթ	Apiaceae	Fruits	The ripe and dried fruit is used against stomach upsets, and as an expectorant, biliary aid, antispasmodic, laxative, and sedative.	[46,63]
Fragaria vesca L.	Yelak antarrayin, getnamori/Ելակ անտառային, գետնամորի	Rosaceae	All parts, especially fruits	Used as an appetizer and to stabilize digestion. It is also used against cholelithiasis, gout, and hepatitis. Decoction of leaves are used against ulcers, gastritis, anemia, hypertension, heart disease, and atherosclerosis.	[45,145]
Fragaria viridis Weston	Yelak kanach kam blrayin/Ելակ կանաչ կամ բլրային	Rosaceae	All parts	Used as an appetizer and to stabilize digestion. It is also used against cholelithiasis, gout, and hepatitis. Decoction of leaves are used against ulcers, gastritis, anemia, hypertension, heart disease, and atherosclerosis.	[92,146]

[a] References are in the manuscript; [b] endemic to the Caucasus.

Table S1 (continued)

**Botanical name**	**Local name** (in Latin/in Armenian)	**Family name**	**Mainly used part**	**Traditional use**	**References**[a]
Frangula alnus Mill.	Bekteni sovorakan, dzhnik/Բեկտենի սովորական, դժնիկ	Rhamnaceae	Trunk's and thick branches's bark, ripe fruits	The ripe fruit is used against liver diseases and their decoction is used against various skin diseases, such as boils and carbuncles.	[49,63]
Fumaria officinalis L.	Tskhabuys deghagortsakan, shantara/Ծխաբույս դեղագործական, շանթարա	Papaveraceae	Aerial parts with flowers	The tincture is used to treat jaundice, biliary tract, and gallbladder diseases, as a diaphoretic, analgesic, and diuretic.	[145,148]
Fumaria schleicheri Soy.-Will.	Tskhabuys Shleykheri/Ծխաբույս Շլեյխերի	Papaveraceae	Aerial parts	In the form of hot wetting, it is used against rheumatism.	[63,149]
Galega officinalis L.	Aytsakhot deghatu, kvoshpegena/Այծախոտ դեղատու, քոշփեգենա	Fabaceae	Flowers and seeds	Decoction of the leaves and seeds is used as an anthelmintic, antipyretic, diuretic, and diaphoretic.	[46,146]
Galium aparine L.	Makardakhot kpchun, vzhvzhuk/Մակարդախոտ կպչուն, վժվժուկ	Rubiaceae	Aerial parts	Used against malignant tumours, liver diseases, as well as against bladder inflammation and swelling. The sap is used against jaundice, cystitis, liver and kidney diseases, and, when applied externally, to treat skin rashes, boils, psoriasis, and other skin diseases.	[45,143]
Galium verum L.	Makardakhot iskakan, makardakhot deghin/Մակարդախոտ իսկական, մակարդախոտ դեղին	Rubiaceae	Aerial parts	Flowers in patch form were used against burns, as a blood clotting. The herb mixed with rose oil eliminates weakness.	[106,146]

[a] References are in the manuscript; [b] endemic to the Caucasus.

Table S1 (continued)

**Botanical name**	**Local name** (in Latin/in Armenian)	**Family name**	**Mainly used part**	**Traditional use**	**References[a]**
Gentiana cruciata L.	Bog khachadzev, odzi degh/Բոզ խաչաձև, օձի դեղ	Gentianaceae	Aerial parts	Used against mucosal inflammation, hepatitis, splenomegaly, tumours.	[100,149]
Geranium collinum Steph. ex Willd.	Khordeni blrayin, aghavnaktuts/Խորդենի բլրային, աղավնակտուց	Geraniaceae	Aerial parts and roots	Decoction used as a styptic and analgesic against haemorrhoids and female ailments, and as an anti-inflammatory, bactericidal against diarrhoea and dysentery.	[49,145]
Geum rivale L.	Shohokram getayin/Շոհոքրամ գետային	Rosaceae	Aerial parts and rhizomes	Decoction of the rhizomes is used against insomnia, headaches, haemorrhoids, and uterine bleeding. Also used as a mouthwash against sore throats and bleeding gums.	[46,65]
Geum urbanum L.	Shohokram kaghakayin/Շոհոքրամ քաղաքային	Rosaceae	Aerial parts and rhizomes	Decoction of the rhizomes is used against insomnia, headaches, haemorrhoids, and uterine bleeding. Also used as a mouthwash against sore throats and bleeding gums.	[56,149]
Glechoma hederacea L.	Getnabaghegh bagheghanman/Գետնաբաղեղ բաղեղանման	Lamiaceae	Aerial parts in a flowering time	Used in teas for gastrointestinal inflammation and bronchitis, and in decoctions against skin diseases.	[65,149]
Glycyrrhiza glabra L.	Matutak merk, marukh/Մատուտակ մերկ, մարուխ	Fabaceae	Roots, rhizomes and underground shoots	The sap of the rhizome is used against nervous disorders, kidney, liver, and bladder diseases.	[45,145]

[a] References are in the manuscript; [b] endemic to the Caucasus.

Table S1 (*continued*)

**Botanical name**	**Local name** (in Latin/in Armenian)	**Family name**	**Mainly used part**	**Traditional use**	**References**[a]
Grossularia reclinata Mill.	Kokrrosheni tekvats/Կոկռոշենի թեքված	Grossulariaceae	Fruits and leaves	An aqueous decoction of the leaves is used against pneumonia, gastritis, enterocolitis, and chronic constipation, while a decoction of the fruit is used as a mild laxative.	[45,148]
Gypsophila bicolor Grossh	Sapnarmat yerkguyn/Սապնարմատ երկգույն	Caryophyllaceae	Aerial parts and roots	An aqueous decoction of the roots is used against gastrointestinal mucous membrane inflammations and cold-related illnesses.	[49,65]
Hemerocallis fulva (L.) L.	Geghorashushan shikakarmir, deghin shu-shan/Գեղորաշուշան շիկակարմիր, դեղին շուշան	Asphodelaceae	Roots	Decoction is used against jaundice, bladder inflamma-tion, difficulty in urination and delayed menstruation. In some cases it is also used as hemostatic.	[56,92]
Heracleum antasiaticum Manden.	Bldrghan, kotsuk/Բլդրղան, կոծուկ	Apiaceae	Roots, fruits, sometimes also flowers	Green water tincture used against inflammation of the liver and gallbladder, gallstones, digestive disorders.	[56,46]
Heracleum transcaucasicum Manden. [b]	Kotsuk andrkovkasyan/Կոծուկ անդրկովկասյան	Apiaceae	Shoots	Green water tincture used against inflammation of the liver and gallbladder, gallstones, digestive disorders.	[30,147]
Herniaria glabra L.	Pvokhatsaghik, aghekhot/Փոխածաղիկ, աղեխոտ	Caryophyllaceae	Aerial parts	Used against kidney pain, kidney, bladder and ureteral disorders.	[49,63]
Hippophae rhamnoides L.	Chichkhan dzhnikanman, laprri/Չիչխան դժնիկանման, լափռի	Elaeagnaceae	Fruits and seeds	Used as an anti-inflammatory, analgesic, and hair-strengthening agent.	[49,143]

[a] References are in the manuscript; [b] endemic to the Caucasus.

Table S1 (*continued*)

**Botanical name**	**Local name** (in Latin/in Armenian)	**Family name**	**Mainly used part**	**Traditional use**	**References**[a]
Humulus lupulus L.	Gayluk sovorakan/Գայլուկ սովորական	Cannabaceae	Cones	Used against gastritis, diarrhea, pneumonia, hair loss, and furuncles.	[51,65]
Hyoscyamus niger L.	Bangi sev, khelarr khot/Բանգի սև, խելառ խոտ	Solanaceae	Leaves	Used externally as an analgesic and sedative.	[46,145]
Hypericum alpestre subsp. *polygonifolium* (Rupr.) Avet. & Takht.	Srohund alpiakan/Սրոհունդ ալպիական	Hypericaceae	Aerial parts	Used as anti-inflammatory against skin diseases.	[46,63,146]
Hypericum eleonorae Jelen.	Srohund Eleonorayin/Սրոհունդ Էլեոնորային	Hypericaceae	Aerial parts	Used as anti-inflammatory against skin diseases.	[30,146]
Hypericum perforatum L.	Srohund khotsvats, arevkurik/Սրոհունդ խոցված, արևքուրիկ	Hypericaceae	Aerial parts	Used against gingivitis, neuralgia, malaria, and several female diseases, as well as against rheumatism, scabies, and gout.	[45,149]
Hippophae rhamnoides L.	Chichkhan sovorakan/Չիչխան սովորական	Elaeagnaceae	Ripe fruits	Decoction of the fruit is used against skin diseases.	[143,149]
Hyssopus angustifolius M.Bieb.	Zopa neghaterev, mshtik/Զոպա նեղատերև, մշտիկ	Lamiaceae	Aerial parts with stems and flowers	The leaves are used as a sedative, and also against gastrointestinal inflammation, asthma, anaemia, and rheumatism.	[49,56]

[a] References are in the manuscript; [b] endemic to the Caucasus.

Table S1 (*continued*)

**Botanical name**	**Local name** (in Latin/in Armenian)	**Family name**	**Mainly used part**	**Traditional use**	**References**[a]
Inula helenium L.	Heghineyi khot/Հեղինեի խոտ	Asteraceae	Roots and rhizomes	Used against hemorrhoids, migraines and itching. Root powder mixed with honey used to clean the stomach when taken internally, against heart and liver diseases.	[145,46]
Juglans regia L.	Ynkuzeni sovorakan/Ընկուզենի սովորական	Juglandaceae	Leaves and immature fruits	The decoction of the leaves is used as a bath for hemorrhoids, it is also used as an anthelmintic. The decoction of the unripe fruit is used against gastritis, diarrhoea, and enterocolitis.	[146,149]
Juniperus oblonga M.Bieb.	Gihi yerkaraterev/Գիհի երկարատերև	Cupressaceae	Fire-apples	Used against epilepsy and itching, and also to open menstruation, and improve hearing.	[51,146]
Lactuca serriola L.	Marrol vayri/Մառոլ վայրի	Asteraceae	Aerial parts and flowers	An aqueous decoction of the green parts is used against coughs, pneumonia, measles, and rheumatic pains.	[51,145]
Lamium album L.	Khul yeghinj, yeghnjamayr/Խուլ եղինջ, եղնջամայր	Lamiaceae	Flowers	Used against respiratory, spleen and kidney problems, and as hemostatic, while in the form of a tea it is used by adults as a sedative and sleeping aid.	[56,148]
Lamium maculatum L.	Khul yeghinj btsavor/Խուլ եղինջ բծավոր	Lamiaceae	Leaves and flowers	The dried and crushed leaves are used to make an aqueous decoction that is used as a hemostatic and an anti-inflammatory.	[49,148]

[a] References are in the manuscript; [b] endemic to the Caucasus.

Table S1 (*continued*)

**Botanical name**	**Local name** (in Latin/in Armenian)	**Family name**	**Mainly used part**	**Traditional use**	**References**[a]
Laser trilobum Borkh. ex Gaertn.	Kaputak yerrabltik/Կապուտակ եռաբլթիկ	Apiaceae	Roots and fruits	Boiled fruit is used against coughs, respiratory infections and intestinal diseases.	[56,147]
Lathraea squamaria L.	Gaghteni tepukavor/Գաղտենի թեփուկավոր	Orobanchaceae	The whole plant	Decoction is used against liver diseases (e.g., hepatitis, cirrhosis), cholecystitis, female, kidney, and gastrointestinal diseases.	[45,65]
Lathyrus pratensis L.	Tapvolorr margagetnayin, kisam/Տափոլոռ մարգագետնային, քիսամ	Fabaceae	Aerial parts	Used as an expectorant against certain respiratory diseases, e.g., cough, bronchitis, pneumonia, inflammation of the lungs.	[147,148]
Lathyrus tuberosus L.	Tapvolorr palarakir, katik/Տափոլոռ պալարակիր, կաթիկ	Fabaceae	Tubers	Decoction of the tubers is used against colitis, dysentery, and diarrhoea.	[45,143,145]
Laurus nobilis L.	Dapni/Դափնի	Lauraceae	Leaves and fruits	Used against rheumatism, earaches, stomach upsets, and sprains.	[45,63]
Lemna minor L.	Jrosp pvokr, dzkan atsveni/Ջրոսպ փոքր, ձկան ածվենի	Araceae	The whole plant	Hot infusion of the plant was used against various tumours. It is used against jaundice, gout, rheumatism, and bone pain.	[46,56]
Leontodon hispidus L.	Arryutsatam manrakhozan/Առյուծատամ մանրախոզան	Asteraceae	Aerial parts, rhizomes, leaves, and flowers	A decoction of green parts is used against diseases of the gallbladder.	[143,144]

[a] References are in the manuscript; [b] endemic to the Caucasus.

Table S1 (continued)

Botanical name	Local name (in Latin/in Armenian)	Family name	Mainly used part	Traditional use	References[a]
Leonurus cardiaca L.	Arryutsagi srtanman/Առյուծագի սրտանման	Lamiaceae	Aerial parts and flowers	Used against some heart, nervous, and gynecological diseases.	[51,65]
Lepidium latifolium L.	Pghpeghik, paron banjar/Պղպեղիկ, պարոն բանջար	Brassicaceae	Aerial parts, flowers, roots, and seeds	The decoction of the leaves is used against wounds and joint pain. The seeds are used externally against rheumatism.	[46,63]
Lepidium campestre (L.) W.T.Aiton	Kotem dashtayin, nvardak/Կոտեմ դաշտային, նվարդակ	Brassicaceae	Aerial parts, flowers, roots, and seeds	The decoction of the leaves is used against wounds and joint pain. The seeds are used externally against rheumatism.	[46,63]
Lilium armenum (Miscz. ex Grossh.) Manden. [b]	Shushan haykakan/Շուշան հայկական	Liliaceae	Leaves with stalk	Used against whooping cough, purulent wounds, burns, leprosy, fungal diseases, mastitis, cystitis.	[119]
Linaria vulgaris Mill.	Ktavakhot sovorakan, vuzhakhot/Կտավախոտ սովորական, վուժախոտ	Plantaginaceae	Aerial parts	An aqueous decoction is used to treat jaundice, kidney and bladder inflammation. A special tea made from the plant is used against headaches, angina, and coughs.	[49,146]
Lithospermum officinale L.	Kakavkrkut deghatu/Կաքավկրկուտ դեղատու	Boraginaceae	The whole plant	Decoction of the roots is used against burns, wounds and swellings caused by insect bites, while decoction of the stems and leaves is used as an antipyretic, as well as against headaches and colds.	[51,56]

[a] References are in the manuscript; [b] endemic to the Caucasus.

Table S1 (*continued*)

**Botanical name**	**Local name** (in Latin/in Armenian)	**Family name**	**Mainly used part**	**Traditional use**	**References**[a]
Lotus corniculatus L.	Yeghjerarrvuyt yeghjravor, nergyun/Եղջերառվույտ եղջրավոր, ներգյուն	Fabaceae	Aerial parts and flowers	Decoction of the green parts is used against respiratory diseases, bronchitis and pneumonia, while decoction of the flowers is used as a soothing and invigorating remedy.	[65,148]
Lythrum salicaria L.	Arenatsaghik sovorakan/Արենածաղիկ սովորական	Lythraceae	Leaves, flowers, stems, and roots	Used against diarrhoea and dysentery. Decoction of the roots and green parts is used for baths for 'weak' children, and fresh leaves are applied to cuts and wounds to heal them quickly.	[49,56]
Malus orientalis Uglitzk.	Khndzoreni arevelyan/Խնձորենի արևելյան	Rosaceae	Fruits and leaves	The ripe fruit is used to improve the digestive system and against vitamin deficiencies. The leaves and fruits are used as an anti-inflammatory, an invigorator, and they help flush excess cholesterol and toxic substances out of the body faster.	[144,146]
Malva neglecta Wallr.	Pipert kloraterev, hatsupanir/Փիփերթ կլորատերև, հացուպանիր	Malvaceae	Aerial parts and leaves	Decoction of the leaves is used against bronchitis, diarrhea, stomach and duodenal ulcers, colitis, and haemorrhoids.	[63,148]
Malva sylvestris L.	Pipert antarrayin, mologh/Փիփերթ անտառային, մոլող	Malvaceae	Aerial parts and leaves	Tea from the leaves and roots is used against stomach and intestinal pain, bladder and ureteral disorders.	[51,63]
Marrubium vulgare L.	Meghratsuts sovorakan/Մեղրածուծ սովորական	Lamiaceae	Aerial parts	Used against inflammation of the teeth and ears, asthma, opens blockages of the liver and spleen.	[145,148]

[a] References are in the manuscript; [b] endemic to the Caucasus.

Table S1 (*continued*)

**Botanical name**	**Local name** (in Latin/in Armenian)	**Family name**	**Mainly used part**	**Traditional use**	**References**[a]
Matricaria chamomilla L.	Yeritsuk deghatnayin, haa-vatsaghik/Երիցուկ դեղատնային, հաավածաղիկ	Asteraceae	Flowers and flower-heads	Used against acute and chronic inflammation of the mucous membranes of the stomach and colitis.	[45,146]
Matricaria matricarioides (Less.) Porter	Yeritsuk yeritsukanman/Երիցուկ երիցուկանման	Asteraceae	Flowers	The tincture is used against toothaches and also externally against skin rashes and wounds.	[65,144]
Melampyrum arvense L.	Kovatsvoren dashtayin/Կովացորեն դաշտային	Orobanchaceae	Aerial parts, flowers and seeds	Tincture of the green parts is used against rheumatism, dizziness, heart problems, headaches, and insomnia.	[46,65]
Melilotus officinalis (L.) Pall.	Isharrvuyt deghatu/Իշառվույտ դեղատու	Fabaceae	Flowers and flower-heads	Used internally and externally against respiratory diseases, insomnia, rheumatoid arthritis, skin abscesses.	[51,65]
Melissa officinalis L.	Patrinj deghatu, trnjkhot/Պատրինջ դեղատու, թրնջխոտ	Lamiaceae	Aerial parts	Aqueous tincture of leaves and shoots is used as an anesthetic, against anemia, headaches, toxemia during pregnancy.	[63,144]
Mentha arvensis L.	Daghdz dashtayin/Դաղձ դաշտային	Lamiaceae	Aerial parts and flowers	Decoction of the plant is used as an appetiser, analgesic, anti-inflammatory, choleretic, it is also often used against gastritis, vomiting, diarrhoea, and dysentery.	[51,146]
Mentha longifolia (L.) L.	Daghdz yerkaraterev, katvakhot/Դաղձ երկարատերև, կատվախոտ	Lamiaceae	Aerial parts	The decoction of the green parts is good against diarrhoea, nausea and stomach aches and the fresh juice helps with snake and scorpion bites.	[45,149]

[a] References are in the manuscript; [b] endemic to the Caucasus.

Table S1 (*continued*)

**Botanical name**	**Local name** (in Latin/in Armenian)	**Family name**	**Mainly used part**	**Traditional use**	**References**[a]
Mentha pulegium L.	Ananukh lvi, lvatsaghik/Անանուխ լվի, լվածաղիկ	Lamiaceae	Leaves and flowers	The leaves and flowers are used as an antiseptic and emollient.	[148]
Menyanthes trifoliata L.	Jrarrvuyt yerraterev/Ջրառվույտ եռատերև	Menyanthaceae	Leaves	The leaves are used against liver and gallbladder diseases, pneumonia, and malaria.	[51,56]
Mespilus germanica L.	Zkerreni sovorakan/Զկեռենի սովորական	Rosaceae	Leaves and fruits	Decoction of the leaves and fruit is used against kidney problems, haemorrhoids, inflammation of the stomach and intestines. Decoction of the leaves is also used as a mouthwash against sore throats.	[51,143]
Morus alba L.	Tteni spitak/Թթենի սպիտակ	Moraceae	Leaves	Used against mild forms of diabetes and skin diseases.	[20, 56]
Myosotis micrantha Pall. ex Lehm.	Anmorruk manratsaghik/Անմոռուկ մանրածաղիկ	Boraginaceae	Aerial parts and roots	The decoction used as an anthelmintic. The juice of the plant is used as a rejuvenator against the elderly, and to improve sexual performance.	[45,146]
Myosotis sylvatica Hoffm.	Anmorruk antarrayin/Անմոռուկ անտառային	Boraginaceae	Aerial parts, roots and flowers	Infusion of green parts or roots is used against eye diseases, e.g., conjunctivitis, ophthalmia.	[46,146]
Myosoton aquaticum Moench.	Papkamaz jrayin/Փափկամազ ջրային	Caryophyllaceae	Aerial parts	The aqueous decoction is used against various skin diseases, eye diseases (e.g., conjunctivitis, blepharitis), and purulent angina.	[46,149]

[a] References are in the manuscript; [b] endemic to the Caucasus.

Table S1 (*continued*)

**Botanical name**	**Local name** (in Latin/in Armenian)	**Family name**	**Mainly used part**	**Traditional use**	**References**[a]
Nasturtium officinale R.Br.	Jrkotem deghatu, arrvikotmuk/Ջրկոտեմ դեղատու, առվիկոտմուկ	Brassicaceae	Aerial parts	Used against gingivitis, diabetes, anaemia, dermatoses, and rheumatism.	[145,46]
Nepeta grandiflora M.Bieb. [b]	Katvadaghdz khoshor-atsaghik/Կատվադաղձ խոշորածաղիկ	Lamiaceae	Aerial parts	The water decoction is used against diseases of the urogenital and digestive system, anaemia, and insomnia.	[45,56,146]
Nigella sativa L.	Sonich/Սոնիճ	Ranunculaceae	Leaves and seeds	The tea made from seeds is used against constipation, stomach and bowel disorders. The decoction of the seeds is used against bronchial asthma, urolithiasis, insomnia, and some skin diseases.	[49,147]
Nymphaea alba L.	Harsnamat spitak, jrashu-shan/Հարսնամատ սպիտակ, ջրաշուշան	Nymphaeaceae	Leaves, flowers, rhizomes and roots	Decoction of the rhizome is used as a hemostatic. The sap of the plant is used to relieve sore throats and coughs, and the roots boiled in sweet juice give relief to pneumonia patients.	[43,150]
Ocimum basilicum L.	Rrehan mshakovi/Ռեհան մշակովի	Lamiaceae	Aerial parts	Used against febrile illnesses, poor digestion, asthma, nausea, gastroenteritis, migraines, and insomnia.	[144,145]
Ononis arvensis L.	Yeznargel dashtayin, ishakhorrn/Եզնարգել դաշտային, իշախոռն	Fabaceae	Roots	Used as a diuretic and antiperspirant, a hemostatic, and as a soothing agent against headaches, rheumatism, chronic skin lesions.	[51,143]

[a] References are in the manuscript; [b] endemic to the Caucasus.

Table S1 (*continued*)

**Botanical name**	**Local name** (in Latin/in Armenian)	**Family name**	**Mainly used part**	**Traditional use**	**References**[a]
Onopordum acanthium L.	Karr pshot, ishakarr/Կառ փշոտ, իշակառ	Asteraceae	Leaves and leafy shoots	Herbal decoction is used against purulent wounds, malignant tumors, pneumonia, and tuberculosis.	[51,65]
Orchis mascula L.	Kholordz arakan, altsatunk/Խոլորձ արական, ալծատունկ	Orchidaceae	Tubers	The decoction is used against diarrhoea, sexual weakness, and is also useful against food and other poisonings.	[145,148]
Orchis palustris Jacq.	Kholordz chahchayin, vorkis/Խոլորձ ճահճային, որքիս	Orchidaceae	Tubers	The decoction is used against diarrhoea, sexual weakness, and is also useful against food and other poisonings.	[45,145]
Origanum vulgare L.	Khnkatsaghik sovorakan, sevakhot/Խնկածաղիկ սովորական, սեվախոտ	Lamiaceae	Aerial parts	Used against pulmonary tuberculosis, rheumatism, epilepsy, as well as headaches and skin lesions.	[51,146]
Orobanche aegyptiaca Pers.	Chragakhot yegiptakan/Ճրագախոտ եգիպտական	Orobanchaceae	Aerial parts	A decoction of the ground part is used against syphilis and is sometimes used against diarrhoea and enterocolitis.	[45,63]
Orobanche lutea Baumg.	Arrvuytayin chragakhot/Առվույտային ճրագախոտ	Orobanchaceae	Stems and flowers	Used as an analgesics.	[46,56]
Orobanche ramosa L.	Chyughavor chragakhot/Ճյուղավոր ճրագախոտ	Orobanchaceae	Stems and flowers	Used as an analgesics.	[46,56]

[a] References are in the manuscript; [b] endemic to the Caucasus.

Table S1 (*continued*)

**Botanical name**	**Local name** (in Latin/in Armenian)	**Family name**	**Mainly used part**	**Traditional use**	**References**[a]
Oxalis acetosella L.	Ttvashuk ttvash, garrnakanjik/Թթվաշուկ թթվաշ, գառնականջիկ	Oxalidaceae	Leaves	Used against atherosclerosis and gallbladder disease, and the leaves, mashed with sugar, are used to make a special refreshing drink that is used as a mild diuretic.	[145,148]
Oxalis corniculata L.	Ttvarrvuyt yeghjravor/Թթվառվույտ եղջրավոր	Oxalidaceae	Leaves	Used against atherosclerosis and gallbladder disease, and the leaves, mashed with sugar, are used to make a special refreshing drink that is used as a mild diuretic.	[145,148]
Padus racemosa (Lam.) Gilib.	Tkheni sovorakan, shan/Թխենի սովորական, սհան	Rosaceae	Fruits	The decoction is used as an anti-inflammatory and astringent, regulating stomach and intestinal activity.	[56,148]
Papaver orientale L. [b]	Kakach arevelyan/Կակաչ արևելյան	Papaveraceae	Aerial parts	Used as an analgesic.	[46,63]
Peucedanum alsaticum L.	Margatsntsu elzasyan/Մարգացնծու էլզասյան	Apiaceae	Roots	Decoction of the roots is used against coughs, headaches, asthma, and fever, as well as toothache as a gargle.	[106,143]
Peucedanum caucasicum (M.Bieb.) K.Koch [b]	Margatsntsu kovkasyan/Մարգացնծու կովկասյան	Apiaceae	Roots	Decoction of the roots is used against coughs, headaches, asthma, and fever, as well as toothache as a gargle.	[45,143]
Peucedanum ruthenicum M.Bieb.	Margatsntsu volgyan, yeritsanuk/Մարգացնծու վոլգյան, երիցանուկ	Apiaceae	Roots	Decoction of the roots is used against coughs, headaches, asthma, and fever, as well as toothache as a gargle.	[149,150]

[a] References are in the manuscript; [b] endemic to the Caucasus.

Table S1 (*continued*)

**Botanical name**	**Local name** (in Latin/in Armenian)	**Family name**	**Mainly used part**	**Traditional use**	**References**[a]
Peganum harmala L.	Spand sovorakan, harmal/Սպանդ սովորական, հարմալ	Nitrariaceae	Aerial parts and fruits	The seeds mixed with wine and other herbs are used against heart problems.	[49,56]
Phlomis tuberosa L. Moench	Bavegh palaravor/Բավեղ պալարավոր	Lamiaceae	Aerial parts and tubers	The decoction is used against pneumonia, dysentery, diarrhoea and fever. Freshly chopped leaves and roots are applied to wounds and sores to accelerate healing.	[49,145]
Phragmites australis (Cav.) Steud.	Yegheg haravayin/Եղեգ հարավային	Poaceae	Rhizomes, leaves, and shoots	Decoction of the rhizomes is used as a diaphoretic, diuretic and choleretic, while a decoction of the roots is used against avitaminosis, during scorpion bite, bone pain, and to remove freckles.	[56,145]
Physalis alkekengi L.	Harsnakhot, bojoj/Հարսնախոտ, բոջոջ	Solanaceae	Roots and fruits	The fruit is used with caution for stomach and intestinal pain. It is used as an analgesic, an anti-inflammatory, and a styptic. An ointment of fruit and olive oil is used externally as a soothing and anti-inflammatory.	[146,148]
Pinus kochiana Klotzsch ex K.Koch	Sochi/Սոճի	Pinaceae	Leaves, buds, gum, and cons	Decoction of the leaves is used against wounds, burns and skin diseases.	[65,46]
Pistacia atlantica Desf.	Khnkeni btaterev/Խնկենի բթատերև	Anacardiaceae	Aerial parts	Used against abdominal discomfort and pain, dyspepsia and peptic ulcer, as a diuretic and stimulant.	[49,147]

[a] References are in the manuscript; [b] endemic to the Caucasus.

Table S1 (*continued*)

**Botanical name**	**Local name** (in Latin/in Armenian)	**Family name**	**Mainly used part**	**Traditional use**	**References**[a]
Pistacia mutica Fisch. et C.A.Mey	Khnkeni btaterev/Խնկենի բթատերև	Anacardiaceae	Leaves and resin	Resin mixed with oil and wax is used as a soothing remedy, a special ointment is made.	[65,144]
Plantago lanceolata L.	Yezan lezu nshtaraterev/Եզան լեզու նշտարատերև	Plantaginaceae	Leaves, seeds, and rarely roots	Used against bronchitis, pulmonary tuberculosis, whooping cough, a number of diseases of the gastrointestinal tract.	[146,149]
Plantago major L.	Yezan lezu, jghakhot/Եզան լեզու, ջղախոտ	Plantaginaceae	Leaves, seeds, and rarely roots	Used against bronchitis, pulmonary tuberculosis, whooping cough, a number of diseases of the gastrointestinal tract.	[51,149]
Polygala anatolica Boiss. et Heldr.	Katnakhot anatoliakan/Կաթնախոտ անատոլիական	Polygalaceae	Roots and leaves	Decoction of the leaves is used against memory loss, male impotence, and as an antipyretic. A decoction of the roots is used against stomach and intestinal disorders, kidney inflammation, diarrhoea, and cystitis.	[56,45]
Polygonatum glaberrimum K.Koch.	Soghomoni knik/Սողոմոնի կնիք	Asparagaceae	The whole plant	Decoction of the rhizome is used against haemorrhoids, acute bronchitis, and pneumonia.	[144,145]
Polygonum aviculare L.	Matitegh chnchghuki/Մատիտեղ ճնճղուկի	Polygonaceae	Aerial parts	Decoction of the aerial parts is used against inflammations of the liver, kidneys, ureters, mucous membranes of the stomach and intestines, while the tea is used against coughs and lung diseases.	[49,56]

[a] References are in the manuscript; [b] endemic to the Caucasus.

Table S1 (continued)

**Botanical name**	**Local name** (in Latin/in Armenian)	**Family name**	**Mainly used part**	**Traditional use**	**References**[a]
Polygonum carneum C.Koch.	Matitegh mandik/Մատիտեղ մանդիկ	Polygonaceae	Roots	Used as an anti-inflammatory against stomach and intestinal ulcers, enteritis and colitis, and as an external application to treat furunculosis and burns.	[45,146]
Polygonum hydropiper L.	Matitegh jrayin/Մատիտեղ ջրային	Polygonaceae	Aerial parts	The fresh herb is used as a mouthwash against inflammation of the mouth, it is also used against fever and sore throat, and when used externally against purulent wounds.	[45,146]
Polygonum persicaria L.	Matitegh deghaterev/Մատիտեղ դեղատերև	Polygonaceae	Aerial parts	The decoction is used as a hemostatic, an anti-inflammatory, diuretic and laxative, and is also used against constipation.	[51,145]
Portulaca oleracea L.	Dandurr banjareghayin, prprem /Դանդուռ բանջարեղային, փրփրեմ	Portulacaceae	Aerial parts and seeds	Used against liver and stomach problems, diarrhoea and ulcers, as well as against dysentery, snake and bee stings.	[45,145]
Potentilla erecta (L.) Raeusch.	Matnuni ughigh, hingterevi/Մատնունի ուղիղ, հինգտերևի	Rosaceae	Aerial parts and rhizomes	Used to treat the gastrointestinal tract, some skin diseases (skin rashes, burns), and inflammation of the mouth. And a decoction of the rhizomes is used as a hemostatic to stop intestinal, uterine and pulmonary bleeding.	[51,56]

[a] References are in the manuscript; [b] endemic to the Caucasus.

Table S1 (continued)

**Botanical name**	**Local name** (in Latin/in Armenian)	**Family name**	**Mainly used part**	**Traditional use**	**References**[a]
Poterium polygamum Waldst. et Kit.	Sevatesuk bazmakogh, jermatak/Սևատեսուկ բազմակող, ջերմատակ	Rosaceae	Aerial parts and roots	The decoction is used as an emollient against purulent wounds. Decoction of the roots is used against pneumonia, diarrhoea, dysentery, and enterocolitis.	[145,147]
Primula veris L.	Gnarbuk garnanayin, ko-vatsntsuk/Գնարբուկ գարնանային, կովացնծուկ	Primulaceae	Rhizome with roots, leaves, and flowers	Root extract is used against coughs, pneumonia, and as a diuretic, laxative, and expectorant. Decoction of the leaves is used against kidney and bladder diseases, and decoction of the flowers is used as a diaphoretic against colds and flu.	[63,148]
Prunella vulgaris L.	Sevaglkhik sovorakan, tsortsoruk/Սևագլխիկ սովորական, ծործորուկ	Lamiaceae	Aerial parts	Tincture of the green mass is used against respiratory diseases (e.g., sore throat, cough, diphtheria), heart and stomach ailments. The plant is also used as an ointment in the treatment of fungal diseases, and a tea made from the flowers is used as an anti-inflammatory.	[46,63]
Prunus armeniaca L. [b]	Tsiran/Ծիրան	Rosaceae	Fruits and resin	Apricot resin is used in the treatment of various skin diseases. The fruit is used against anaemia, cardiovascu-lar, and kidney diseases.	[20,49]
Prunus divaricata Le-deb.	Saloreni chrrvats, alucha/Սալորենի չռված, ալուչա	Rosaceae	Flowers, fruits, roots, and branch bark	The fruit is used as an appetizing and as a digestive aid.	[51,143]

[a] References are in the manuscript; [b] endemic to the Caucasus.

Table S1 (*continued*)

Botanical name	Local name (in Latin/in Armenian)	Family name	Mainly used part	Traditional use	References[a]
Prunus spinosa L.	Saloreni pshavor, sev salor/Սալորենի փշավոր, սև սալոր	Rosaceae	Flowers and fruits	Fruits are used fresh or cooked as an anti-inflammatory and tonic against intestinal disorders, hoarseness, and cough.	[51,143]
Pulmonaria dacica Simonk.	Meghruk/Մեղրուկ	Boraginaceae	Leaves and aerial parts with flowers	Used as an emollient, styptic, and antiseptic.	[92,150]
Pulsatilla albana (Steven) Bercht. & J.Presl.	Knakhot/Քնախոտ	Ranunculaceae	Aerial parts, in some cases even the roots	Used as a sedative, analgesic, anti-inflammatory, and hypnotic.	[144,145]
Punica granatum L.	Nrrneni sovorakan, nrrni/Նռնենի սովորական, նռնի	Lythraceae	Roots, fruits, and trunk barks	The bark of the fruit is used as an antiseptic against stomach and intestinal inflammation, and dysentery.	[144,148]
Pyrus caucasica Fed.[b]	Tandzeni kovkasyan/Տանձենի կովկասյան	Rosaceae	Fruits and leaves	Decoction of the leaves is used against angina and sore throats, the sap of the fruit quenches thirst and improves stomach function, and the seeds have an anthelmintic effect.	[45,148]
Quercus araxina (Trautv.) Grossh.	Kaghni araksyan/Կաղնի արաքսյան	Fagaceae	Fruits and leaves	The fruit is beaten off and placed on the bites of poisonous insects and reptiles to relieve swelling.	[145]
Quercus iberica Stev.[b]	Kaghni vratsakan/Կաղնի վրացական	Fagaceae	Fruits and leaves	The fruit is beaten off and placed on the bites of poisonous insects and reptiles to relieve swelling.	[45,143]

[a] References are in the manuscript; [b] endemic to the Caucasus.

Table S1 (*continued*)

**Botanical name**	**Local name** (in Latin/in Armenian)	**Family name**	**Mainly used part**	**Traditional use**	**References**[a]
Quercus macranthera Fisch. & C.A.Mey.	Kaghni arevelyan, khozakaghni/Կաղնի արևելյան, խոզակաղնի	Fagaceae	Fruits and leaves	The fruit is beaten off and placed on the bites of poisonous insects and reptiles to relieve swelling.	[45,143]
Quercu robur L.	Kaghni amarrayin/Կաղնի ամառային	Fagaceae	Fruits and leaves	The fruit is beaten off and placed on the bites of poisonous insects and reptiles to relieve swelling.	[51,143]
Raphanus raphanistrum L.	Sut boghk, ktarr/Սուտ բողկ, քթառ	Brassicaceae	The whole plant	Used against goitre, urolithiasis and jaundice. Nowadays, the plant is used as an appetiser and gastrointestinal remedy.	[46,148]
Reseda lutea L.	Hapruk deghin/Հափրուկ դեղին	Resedaceae	Aerial parts, leaves, and roots	Decoction is used as an anthelmintic, diaphoretic, and diuretic.	[56,149]
Rhamnus cathartica L.	Dzhnik lutsoghakan, hakri /Դժնիկ լուծողական, հակրի	Rhamnaceae	Rip fruits	Decoction of the fruit is used as a laxative, in the treatment of jaundice, hemorrhoids, gastritis. Fruit sap is also used against skin diseases.	[49,63]
Rheum luteola L.	Khavartsil/Խավարծիլ	Polygonaceae	Rhizomes with roots	Used as a choleretic, mild laxative, and anti-inflammatory against haemorrhoids and constipation.	[46,148]

[a] References are in the manuscript; [b] endemic to the Caucasus.

Table S1 (*continued*)

**Botanical name**	**Local name** (in Latin/in Armenian)	**Family name**	**Mainly used part**	**Traditional use**	**References**[a]
Rheum palmatum L.	Khavartsil deghatu/Խավարծիլ դեղատու	Polygonaceae	Rhizomes with roots	Used as a choleretic, mild laxative, and anti-inflammatory against haemorrhoids and constipation.	[65,148]
Rhinanthus minor L.	Aklorabbuk pvokr/Աքլորաբբուկ փոքր	Orobanchaceae	Aerial parts	Decoction of the green part is used against alcoholism, arrhythmia, fever, headaches, as well as venereal diseases. The dried and crushed leaves are used externally against purulent wounds.	[49,56]
Rhinanthus pectinatus (Behrend.) Vass.	Aklorabbuk sanravor/Աքլորաբբուկ սանրավոր	Orobanchaceae	Aerial parts	Decoction of the green part is used to treat alcoholism, arrhythmia, fever, headaches, as well as venereal diseases. The dried and crushed leaves are used externally to treat purulent wounds.	[49,65]
Rhus coriaria L.	Drakhtatsarr/Դրախտածառ	Anacardiaceae	Leaves and fruits	An aqueous tincture of the leaves, an alcoholic extract or a decoction are used against diabetes. The preparations are used against inflammation of the eyes and mouth.	[65,148]
Ribes alpinum L.	Hagharjeni alpiakan/Հաղարջենի ալպիական	Grossulariaceae	Leaves, fruits, and buds	Decoction of the leaves is used against rheumatism and gout, while the fruit is used against vitamin deficiencies.	[45,143]
Ribes armenum Pojark. (*Ribes nigrum* L.) [b]	Hagharjeni haykakan/Հաղարջենի հայկական	Grossulariaceae	Leaves, fruits, and buds	Fresh fruit is used to make a syrup that is used to change the aftertaste of many medicines.	[30]

[a] References are in the manuscript; [b] endemic to the Caucasus.

Table S1 (*continued*)

**Botanical name**	**Local name** (in Latin/in Armenian)	**Family name**	**Mainly used part**	**Traditional use**	**References**[a]
Ribes biebersteinii Berland.	Hagharjeni Bibershteyni/Հաղարջենի Բիբերշտեյնի	Grossulariaceae	Leaves, fruits, and buds	Decoction of the leaves is used against rheumatism and gout, while the fruit is used against vitamin deficiencies.	[56,150]
Rosa canina L.	Masreni shan, shnavard/Մասրենի շան, շնավարդ	Rosaceae	Fruits, roots, and flowers	Decoction of roots is used in the treatment of hemorrhoids in a bath. The sap of the fruit is used against eye and ear diseases, as well as headaches and toothaches.	[106,145]
Rosa corymbifera Borkh.	Masreni vahanakir/Մասրենի վահանակիր	Rosaceae	Petals	Used against liver diseases.	[65,144]
Rosa sosnovskyana Tam. [b]	Masreni andrkovkasyan/Մասրենի անդրկովկասյան	Rosaceae	Fruits	Used against liver diseases.	[30]
Rosa spinosissima L.	Masreni arratapush/Մասրենի առատափուշ	Rosaceae	Petals	Used against heart diseases.	[45,146]
Rubia tinctorum L.	Toron nerkatu, gunarmat/Տորոն ներկատու, գունարմատ	Rubiaceae	Rhizomes and roots	Used in the form of decoctions or infusions mixed with mint against jaundice.	[65,148]

[a] References are in the manuscript; [b] endemic to the Caucasus.

Table S1 (*continued*)

**Botanical name**	**Local name** (in Latin/in Armenian)	**Family name**	**Mainly used part**	**Traditional use**	**References**[a]
Rubus armeniacus Focke [b]	Morreni haykakan/Մոռենի հայկական	Rosaceae	Leaves, fruits and roots	Used against leprosy, inflammation of the digestive tract, plague, burns.	[45,143]
Rubus caesius L.	Mosheni sovorakan, moshi/Մոշենի սովորական, մոշի	Rosaceae	Leaves and fruits	Tea made from fruits and leaves is used as a diaphoretic and antipyretic against colds.	[49,148]
Rubus idaeus L.	Antaramori, aznvamori/Անտառամորի, ազնվամորի	Rosaceae	Fruits and flowers	The fruit tincture is used against influenza, chronic rheumatism, scurvy, anemia and gastric diseases. The fruit was cooked with honey and used against measles. The flower tincture was used against snakebite in the form of compress.	[51,143]
Rubus saxatilisn L.	Mosheni karayin, karamori/Մոշենի քարային, քարամորի	Rosaceae	Fruits, leaves and stems	Decoction of the whole plant is used to strengthen hair and get rid of dandruff. Decoction of the leaves is used against gastrointestinal and female ailments, and the prepared tea is used to soothe the nerves.	[45,143]
Rubus takhtadjanii Mulk. [b]	Aznvamori haykakan/Ազնվամորի հայկական	Rosaceae	Fruits	Tea made from fruits and leaves is used as a diaphoretic and antipyretic against colds.	[30,143]

[a] References are in the manuscript; [b] endemic to the Caucasus.

Table S1 (*continued*)

**Botanical name**	**Local name** (in Latin/in Armenian)	**Family name**	**Mainly used part**	**Traditional use**	**References**[a]
Rumex alpinus L.	Aveluk alpiakan, trntjuk alpiakan/Ավելուկ ալպիական, թրնթջուկ ալպիական	Polygonaceae	Roots and leaves	Used against melancholy, gastrointestinal disorders, infectious diseases, metabolic disorders.	[56,148]
Rumex crispus L.	Aveluk gangur, trntjuk gangur/Ավելուկ գանգուր, թրնթջուկ գանգուր	Polygonaceae	Aerial parts with leaves and flowers	Decoction of the roots is used against coughs, bronchitis, and upper respiratory tract diseases. Fresh leaves or their sap are used as an appetite suppressant, a hemostatic, and a diuretic.	[49,148]
Sagittaria sagittifolia L.	Netaxot netaxotanman/Նետախոտ նետախոտանման	Alismataceae	Rhizomes and leaves	Used as an analgesic and an astringent.	[46,65,146]
Salicornia europaea L.	Aghabuys yevropakan, momik/Աղաբույս եվրոպական, մոմիկ	Amaranthaceae	Aerial parts	The decoction is used against diseases of the urogenital system.	[21,149]
Salix alba L.	Urri, urreni spitak/Ուռի, ուռենի սպիտակ	Salicaceae	Trunk and branch bark, leaves and roots	Used against diseases of the spleen, gastric mucosa, inflammation of the liver and kidneys. The smell and sap of the flowers is used against headaches, the resin of the tree improves eyesight.	[143,144]
Salix caprea L.	Urreni pshik, aytsurri/Ուռենի փշիկ, այծուռի	Salicaceae	Leaves and roots	Used against diseases of the spleen, gastric mucosa, inflammation of the liver and kidneys. The smell and sap of the flowers is used against headaches, the resin of the tree improves eyesight.	[143,144]

[a] References are in the manuscript; [b] endemic to the Caucasus.

Table S1 (*continued*)

**Botanical name**	**Local name** (in Latin/in Armenian)	**Family name**	**Mainly used part**	**Traditional use**	**References**[a]
Salix cinerea L.	Urreni mokhraguyn/Ուռենի մոխրագույն	Salicaceae	Leaves and roots	Used against diseases of the spleen, gastric mucosa, inflammation of the liver and kidneys. The smell and sap of the flowers is used against headaches, the resin of the tree improves eyesight.	[143,144]
Salvia officinalis L.	Yeghespak deghatu/Եղեսպակ դեղատու	Lamiaceae	Aerial parts	Used against kidney pain, bladder pain, cough, weakness.	[51,145]
Salvia sclarea L.	Yeghespak mshkynkuzayin, sherepanuk/Եղեսպակ մշկընկուզային, շերեփանուկ	Lamiaceae	Aerial parts	Decoction of the leaves is used against kidney and bladder diseases, it has a styptic effect, and relieves skin rashes.	[49,63]
Sambucus ebulus L.	Kttkeni khotayin, tztpaghuk/Կտտկենի խոտային, տզտպաղուկ	Adoxaceae	Aerial parts, fruits, and roots	Decoction of the leaves is used against inflammation of the urinary tract, and the fruit is used as a laxative and diuretic.	[45,143]
Sambucus nigra L.	Kalantashi tsarr, tantrveni/Քալանթաշի ծառ, թանթրվենի	Adoxaceae	The whole plant	Flowers are used as sudorific and diuretic are useful in uremia.	[51,65]
Saponaria officinalis L.	Ocharrabuys, prprik/Օճառաբույս, փրփրիկ	Caryophyllaceae	Roots	Used against oral mucous membrane ulcers, urinary and menstrual retention, visual disturbances.	[21,65]
Satureja hortensis L.	Kortin partezayin, tsitron/Կորթին պարտեզային, ծիթրոն	Lamiaceae	Aerial parts	Used against flu, cough, headache, migraine, dizziness, irregular heartbeat.	[49,63]

[a] References are in the manuscript; [b] endemic to the Caucasus.

Table S1 (*continued*)

**Botanical name**	**Local name** (in Latin/in Armenian)	**Family name**	**Mainly used part**	**Traditional use**	**References**[a]
Scorzonera latifolia (Fisch. et Mey.) DC.	Sindz laynaterev, katnaberik/Սինձ լայնատերև, կաթնաբերիկ	Asteraceae	Roots and leaves	The local population dries the juice extracted from the roots and uses it as chewing gum, calling it 'mountain gum'. Resin, derived from the roots and green parts, was used to whiten teeth and to strengthen the gums.	[49,146]
Sedum acre L.	Tantrrnik darrn, gortnukabuys/Թանթռնիկ դառն, գորտնուկաբույս	Crassulaceae	Aerial parts	The sap is used against vitamin deficiencies, epilepsy, and anaemia. It is also used externally to remove warts and papillomas, and against purulent wounds and ulcers.	[46,143]
Sedum album L.	Spitak tantrrnik/Սպիտակ թանթռնիկ	Crassulaceae	Aerial parts	The sap is used against vitamin deficiencies, epilepsy, and anaemia. It is also used externally to remove warts and papillomas, and against purulent wounds and ulcers.	[65,148]
Senecio rhombifolia (Adam) Sch. Bip.	Halevoruk sheghankyunadzev/Հալևորուկ շեղանկյունաձև	Asteraceae	The whole plant	A decoction of the roots was used against various tumours and infertility in women.	[20,147]
Sinapis arvensis L.	Mananekh vayri, akhuna/Մանանեխ վայրի, ախունա	Brassicaceae	Seeds	Used as an appetizer and anthelmintic	[51,145]
Sisymbrium officinale (L.) Scop.	Khozaknchit deghatu, aghbagh-buk/Խոզակնճիթ դեղատու, աղբաղբուկ	Brassicaceae	Aerial parts and sometimes roots	Sap obtained from fresh leaves is used as an anthelmintic, remedy against gingivitis, expectorant, and diuretic.	[148,149]

[a] References are in the manuscript; [b] endemic to the Caucasus.

Table S1 (*continued*)

**Botanical name**	**Local name** (in Latin/in Armenian)	**Family name**	**Mainly used part**	**Traditional use**	**References**[a]
Solanum dulcamara L.	Morm kaghtsradarrn/Մորմ քաղցրադառն	Solanaceae	Shoots with leaves, flowers, and fruits	Decoction of the leaves and shoots is used against asthma, bladder and urinary tract infections, it is also used to regulate metabolism, against headaches, and epilepsy.	[49,65]
Solanum nigrum L.	Morm sev, katvakhaghogh/Մորմ սև, կատվախաղող	Solanaceae	Shoots with leaves and fruits	The decoction is used as an antipyretic, anti-inflammatory, and diuretic against bladder spasms and stomach pains.	[20,143]
Sophora japonica (L.) Schott	Sofora chaponakan, sofora/Սոֆորա ճապոնական, սոֆորա	Fabaceae	Flower-buds and fruits	Decoction of the fruit is used against burns, various ulcers, and purulent wounds.	[21,149]
Sorbus aucuparia L.	Aroseni, tstikhndzor/Արոսենի, ծտիխնձոր	Rosaceae	Fruits	Tincture of the fruit is used as a soft laxative, biliary, and diuretic.	[51,143]
Sorbus hajastana Gabrieljan [b]	Haykakan aroseni/Հայկական արոսենի	Rosaceae	Fruits	Tincture of the fruit is used as a soft laxative, biliary, and diuretic.	[30,143]
Sorghum halepense (L.) Pers.	Molasorgo, halepi khot/Մոլասորգո, հալեպի խոտ	Poaceae	Seeds and roots	Decoction of roots and rhizomes is used with caution against gout and rheumatism, while decoction of seeds is used as a diuretic, and as a general restorative.	[51,146]

[a] References are in the manuscript; [b] endemic to the Caucasus.

Table S1 (*continued*)

**Botanical name**	**Local name** (in Latin/in Armenian)	**Family name**	**Mainly used part**	**Traditional use**	**References**[a]
Stachys palustris L.	Abeghakhot chahchayin, yeznakhot/Աբեղախոտ ճահճային, եզնախոտ	Lamiaceae	Aerial parts	Used in the treatment of female illnesses in the postpartum period. Decoction of the green mass is used against irregular menstruation and the decoction is used as a rubbing and bath against inflammatory uterine diseases and other female ailments.	[46,143]
Stellaria media (L.) Vill.	Astghik mijak, jrjruk/Աստղիկ միջակ, ջրջրուկ	Caryophyllaceae	The whole plant or only aerial parts	A decoction obtained from the whole plant is used as an irrigation against ulcers and purulent wounds. The decoction of aerial parts is used to regulate the gastrointestinal tract and improve nervous system and heart function.	[149]
Symphytum asperum Lepech.	Hayvaz, meghratsuts/Հայվազ, մեղրածուծ	Boraginaceae	Roots and rhizomes	The decoction of the roots is used as an appetizer. Decoction of the plant or infusion of dried roots is also used as a poultice against purulent wounds, bruises, and fractures.	[46,146]
Tamus communis L.	Knkabuys, kuysarmat, sev loshtak/Կնքաբույս, կույսարմատ, սև լոշտակ	Dioscoreaceae	Rhizomes	Using hot poultices, massages or rubbing, the sap or decoction is used against sciatica, rheumatism, gout, and arthritis.	[65,148]
Tanacetum coccineum (Willd.) Grierson	Lvatsaghik mug karmir, tarkavan/Լվածաղիկ մուգ կարմիր, տարկավան	Asteraceae	Inflorescences	Decoction of the flower baskets is used as an anthelmintic, and against wounds and ulcers.	[56,146]

[a] References are in the manuscript; [b] endemic to the Caucasus.

Table S1 (*continued*)

**Botanical name**	**Local name** (in Latin/in Armenian)	**Family name**	**Mainly used part**	**Traditional use**	**References**[a]
Tanacetum vulgare L.	Lvatsaghik sovorakan, susambar/Լվածաղիկ սովորական, սուսամբար	Asteraceae	Inflorescences	The tincture is used as an anthelmintic, anti-inflammatory, choleretic, and antiseptic.	[56,146]
Taraxacum officinale F.H.Wigg.	Krrabanjar/Կռաբանջար	Asteraceae	The whole plant	Used to "purify the blood", has the ability to regulate digestion, eliminate jaundice, and regulate sleep.	[51,63]
Taxus baccata L.	Keni hataptghayin, geghdzi/Կենի հատապտղային, գեղձի	Taxaceae	Leaves	Decoction of the leaves is only used externally against colds, asthma, epilepsy, kidney, and liver diseases.	[143,146]
Teucrium polium L.	Lerdakhot aleher, mariam khot/Լերդախոտ ալեհեր, մարիամ խոտ	Lamiaceae	The whole plant	Used as an anthelmintic and diuretic, the juice is used against diseases of the liver and spleen.	[144,145]
Teucrium scordioides Schreb.	Lerdakhot skhtorayin/Լերդախոտ սխտորային	Lamiaceae	The whole plant	Fresh or dried herb mixed with vinegar was used against joint pains, and a mixture with honey was used against malignant ulcers and skin diseases. Also used in the form of wettings and baths against purulent wounds, boils, and eye inflammation.	[144,145]
Thalictrum foetidum L.	Gndzmndzuk/Գնձմնձուկ	Ranunculaceae	Aerial parts and leaves	Used in limited quantities as a sedative in neuroses and as a general tonic against gall bladder, liver and kidney diseases.	[146,148]

[a] References are in the manuscript; [b] endemic to the Caucasus.

Table S1 (*continued*)

**Botanical name**	**Local name** (in Latin/in Armenian)	**Family name**	**Mainly used part**	**Traditional use**	**References**[a]
Thalictrum minus L.	Khmrakhot pvokr, gndzmndzuk pvokr/Խմրախոտ փոքր, գնձմնձուկ փոքր	Ranunculaceae	Aerial parts and roots	The root decoction is used against jaundice, headaches, epilepsy and metabolic disorders. While the herbal decoction is used against acute infectious diseases such as measles, smallpox, diphtheria, malaria.	[146,148]
Thymus collinus Bieb.	Urts blrakayin/Ուրց բլրակային	Lamiaceae	Aerial parts	Used as an appetizer, helps with toothache when eating with food, strengthens eyesight, and regulates breathing.	[65,150]
Thymus kotschianus Boiss. et Hohen.	Urts Kochii/Ուրց Կոչիի	Lamiaceae	Aerial parts	Used as an appetizer, helps with toothache when eating with food, strengthens eyesight, and regulates breathing.	[63,106]
Thymus serpyllum L.	Urts soghatsvogh, vayri tsotrin, mesh/Ուրց սողացող, վայրի ծոթրին, մեշ	Lamiaceae	Aerial parts	Decoction is used as an antiseptic and antispasmodic to improve digestion, and treat oral inflammation.	[49,148]
Thymus transcaucasicus Ronn. [b]	Urts andrkovkasyan/Ուրց անդրկովկասյան	Lamiaceae	Aerial parts	Decoction is used as an antiseptic and antispasmodic to improve digestion, and treat oral inflammation.	[30,106]
Tilia caucasica Rupr.[b]	Loreni kovkasyan/Լորենի կովկասյան	Malvaceae	Flower-heads	Decoction is used against gastric and metabolic disorders and haemorrhoids, and hot decoction of the flowers as a mouthwash is used against throat and mouth cavity.	[51,143]

[a] References are in the manuscript; [b] endemic to the Caucasus.

Table S1 (*continued*)

**Botanical name**	**Local name** (in Latin/in Armenian)	**Family name**	**Mainly used part**	**Traditional use**	**References**[a]
Tilia cordata Mill.	Loreni srtadzev, tmbi/Լորենի սրտաձև, թմբի	Malvaceae	Flower-heads	Decoction is used against gastric and metabolic disorders and haemorrhoids, and hot decoction of the flowers as a mouthwash is used against throat and mouth cavity.	[51,143]
Tribulus terrestris L.	Tatash prrvogh/Տատաշ փռվող	Zygophyllaceae	Leaves and stems	Used as an antiseptic, anti-inflammatory, performance, and sexual health enhancer.	[146,145]
Trifolium pratense L.	Yereknuk margagetnayin, akhlakhndzor/Երեքնուկ մարգագետնային, ախլախնձոր	Fabaceae	Leaves and flower-heads	Decoction of leaves is used against chronic rheumatism, fever, headache, cholecystitis, gastritis, while decoction of flower heads is used against malaria, goiter, rheumatism, and anemia.	[49,145]
Trifolium repens L.	Yereknuk soghatsvogh, yereknuk spitak/Երեքնուկ սողացող, երեքնուկ սպիտակ	Fabaceae	Leaves and flower-heads	Decoction of leaves is used against chronic rheumatism, fever, headache, cholecystitis, gastritis, while decoction of flower heads is used against malaria, goiter, rheumatism, and anemia.	[49,145]
Trigonella foenum-graecum L.	Hatshamem, shambala/Հացհամեմ, շամբալա	Fabaceae	Seeds	Decoction of the seeds is used as an expectorant against of bronchitis and severe coughing. The seeds are also used as an antihelmintic and to kill human parasites (such as fleas and lice).	[63,146]

[a] References are in the manuscript; [b] endemic to the Caucasus.

Table S1 (*continued*)

**Botanical name**	**Local name** (in Latin/in Armenian)	**Family name**	**Mainly used part**	**Traditional use**	**References**[a]
Tussilago farfara L.	Tatrak, khochkorik/Տատրակ, խոճկորիկ	Asteraceae	Leaves	Leaves placed on blains, carbuncles, and other types of abscesses to further accelerate the inflammatory process.	[145,149]
Typha angustifolia L.	Keron neghaterev/Կերոն նեղատերև	Typhaceae	Rhizomes and leaves	Decoction of the rhizomes as a gargle is used against stomatitis and gingivitis, while dried and ground flowers mixed with lard are used against haemorrhoids and inflammation of the rectum.	[49,143]
Typha latifolia L.	Keron laynaterev/Կերոն լայնատերև	Typhaceae	Rhizomes and leaves	Decoction of the rhizomes as a gargle is used against stomatitis and gingivitis, while dried and ground flowers mixed with lard are used against haemorrhoids and inflammation of the rectum.	[145,146]
Urtica dioica L.	Yeghinj yerktun, gyazgyazuk/Եղինջ երկտուն, գյազգյազուկ	Urticaceae	Leaves, flowers, and roots	The sap of the plant is mixed with beer and used to relieve cough and toothache.	[51,56]
Urtica urens L.	Yeghinj ayrogh, dardahan/Եղինջ այրող, դարդահան	Urticaceae	Aerial parts, seeds, and roots	A decoction of the roots is used to regulate the stomach and treat headaches, whooping cough and pneumonia, while a decoction of the seeds is used against sexual impotence.	[65,146]
Vaccinium myrtillus L.	Hapalaseni mrtenakan/Հապալասենի մրտենական	Ericaceae	Leaves and fruits	Dried fruit tea is used against chronic and acute diarrhoea, especially for children.	[65,146]

[a] References are in the manuscript; [b] endemic to the Caucasus.

Table S1 (*continued*)

**Botanical name**	**Local name** (in Latin/in Armenian)	**Family name**	**Mainly used part**	**Traditional use**	**References**[a]
Valeriana officinalis L.	Katvakhot deghatu/Կատվախոտ դեղատու	Caprifoliaceae	Rhizome and roots	Widely used against migraines, heart pain, nervous exhaustion.	[51,56,146]
Veratrum album L.	Keghdz ghandzlamer spitak, jok/Կեղծ ղանձլամեր սպիտակ, ջոք	Melanthiaceae	Rhizomes with roots	Decoction of dried roots is used against skin diseases, rheumatic and neurological pains, and sometimes decoction of the roots is used as an anthelmintic.	[46,65]
Veratrum lobelianum Bernh.	Ghandzlamer/Ղանձլամեր	Melanthiaceae	Rhizomes with roots	Decoction of dried roots is used against skin diseases, rheumatic and neurological pains, and sometimes decoction of the roots is used as an anthelmintic.	[46,63]
Verbascum phlomoides L.	Khrrndat deghatu, yeznazgi/Խռնդատ դեղատու, եզնազգի	Scrophulariaceae	Leaves and flowers	Flower decoction with milk, used against pulmonary tuberculosis, abdominal cramps, throat diseases, and bad breath. Fresh leaf decoction is used against rheumatism, arthritis, and nerve pain.	[46,46]
Verbascum speciosum Schrad.	Khrrndat hrashali, archichuk/Խռնդատ հրաշալի, արճիճուկ	Scrophulariaceae	Leaves and flowers	Flower decoction with milk, used against pulmonary tuberculosis, abdominal cramps, throat diseases, and bad breath. Fresh leaf decoction is used against rheumatism, arthritis, and nerve pain.	[150]

[a] References are in the manuscript; [b] endemic to the Caucasus.

Table S1 (*continued*)

**Botanical name**	**Local name** (in Latin/in Armenian)	**Family name**	**Mainly used part**	**Traditional use**	**References**[a]
Verbena officinalis L.	Aghavnich deghagortsakan, divamushk/Աղավնիճ դեղագործական, դիվամուշկ	Verbenaceae	Leaves, flowers, sometimes seeds	Decoction of the leaves is used to strengthen the roots of gums and teeth, and against ulcers and wounds. The plant is also used against headaches and toothaches.	[145]
Veronica beccabunga L.	Berenike hoskavor, dziu jrkotem/Բերենիկե հոսքավոր, ձիու ջրկոտեմ	Plantaginaceae	Aerial parts and flowers	Used against sore throats and colds and as a gargle for inflammation of the mucous membranes in the pharynx and oral cavity.	[46,63,146]
Viburnum opulus L.	Brrnchi sovorakan/Բռնչի սովորական	Adoxaceae	The bark of branches and stems, and ripe fruits	Used against diarrhoea, sore throat, and gums.	[46,148]
Viburnum lantana L.	Gerimasti/Գերիմաստի	Adoxaceae	Stems and fruits	Used against diarrhoea, sore throat and gums.	[46,148]
Vinca minor L.	Kusatsaghik pvokr/Կուսածաղիկ փոքր	Apocynaceae	Aerial parts, often leaves	Used to stop nasal, uterine, and intestinal bleeding. Used as an anti-inflammatory against sore throats, mouths, wounds and toothaches.	[46,65]
Viola arvensis Murray	Manushak dashtayin/Մանուշակ դաշտային	Violaceae	Aerial parts and roots	Tea prepared from the ground part is used as a nerve relaxant. On the other hand, the plant is also used as a diuretic in the treatment of kidney, bladder, and cholelithiasis.	[46,144]

[a] References are in the manuscript; [b] endemic to the Caucasus.

Table S1 (*continued*)

**Botanical name**	**Local name** (in Latin/in Armenian)	**Family name**	**Mainly used part**	**Traditional use**	**References**[a]
Viola odorata L.	Manushak buravet/Մանուշակ բուրավետ	Violaceae	Aerial parts and roots	Decoction is used as an expectorant for treating the respiratory tract and adenia.	[46,144]
Viola tricolor L.	Manushak yerraguyn/Մանուշակ եռագույն	Violaceae	Aerial parts and roots	Decoction is used as an expectorant for treating the respiratory tract and adenia.	[49,65]
Viscum album L.	Mghamuch spitak/Մղամուճ սպիտակ	Santalaceae	Flowers and shoots	In the form of decoction or tincture herb is used against abdominal pain, epilepsy, hypochondria.	[49,63]
Vitis sylvestris C.C.Gmel.	Khaghogh antarrayin, vayri khag-hogheni/Խաղող անտառային, վայրի խաղողենի	Vitaceae	Ripe fruits	Used against liver and kidney diseases.	[46,65]
Xanthium strumarium L.	Darrnapush papuk/Դառնափուշ փափուկ	Asteraceae	Aerial parts during flowering, fruits, and roots	Decoction of the seeds and roots is used against diarrhoea and dysentery, rheumatism, thyroid, and bladder diseases.	[46,143]
Xeranthemum squarrosum Boiss.	Anmerruk chrrvats, chvorabuys/Անմեռուկ չռված, չորաբույս	Asteraceae	Leaves and stems	An aqueous infusion is used against uterine bleeding, nerve and heart problems.	[20,56]
Ziziphus jujuba Mill.	Unab sovorakan, unabi/Ունաբ սովորական, ունաբի	Rhamnaceae	Seeds, leaves and roots bark	Used as an expectorant, tonic and laxative.	[145,147]
Zygophyllum fabago L.	Zugaterev sovorakan, dziapo-pok/Զուգատերև սովորական, ձիապոպոք	Zygophyllaceae	Roots, stems, leaves, flowers, and buds	Decoction of the roots is used as an emollient in the treatment of various skin diseases.	[21,46]

[a] References are in the manuscript; [b] endemic to the Caucasus.

Supplementary Information Part 2

Traditionally used medicinal plants of Armenia

[1] Pharmazeutisches Institut, Abteilung Pharmazeutische Biologie, Christian-Albrechts-Universität zu Kiel, Gutenbergstraße 76, 24118 Kiel, Germany; ayvazyanarpine95@gmail.com (A. Ayvazyan), czidorn@pharmazie.uni-kiel.de (C. Zidorn)

[2] Division of Pharmaceutical Biotechnology, Department of Pharmaceutical Biology and Biotechnology, Wroclaw Medical University, Borowska 211, 50-556 Wrocław, Poland

* Correspondence: czidorn@pharmazie.uni-kiel.de; Tel.: +49 431 880-1139

* Corresponding author. Pharmazeutisches Institut, Abteilung Pharmazeutische Biologie, Christian-Albrechts-Universität zu Kiel, Gutenbergstraße 76, 24118 Kiel, Germany

E-mail address: czidorn@pharmazie.uni-kiel.de (C. Zidorn)

Table S2. Common names of plant taxa used in Armenian traditional medicine.

Botanical name	Common name
Achillea millefolium L.	Yarrow
Acorus calamus L.	Sweet flag/ sedge
Adonis aestivalis L.	Summer pheasant's-eye
Adonis vernalis L.	Spring pheasant's eye
Aesculus hippocastanum L.	Horse chestnut
Agrimonia eupatoria L.	Agrimony
Agropyron repens (L.) P. Beauv.	Rusty grass
Alchemilla smirnovii Juz.	Lady's mantle
Alhagi persarum Boiss. et Buhse	Manna trees
Alhagi pseudalhagi (Bieb.) Fisch.	Camelthorns
Alkanna orientalis (L.) Boiss.	Alcanet
Alliaria petiolata (Bieb.) Cavara et Grande	Garlic mustard
Allium ursinum L.	Wild garlic
Allium atroviolaceum Boiss	Dark-violet garlic/onion
Allium cepa L.	Onion
Althaea officinalis L.	Marsh-mallow/white mallow
Althaea armeniaca Ten.	Armenian marshmallow
Amaranthus retroflexus L.	Rough pigweed
Amygdalus fenzliana (Fritsch) Lipsky	Bitter almond
Anagallis arvensis L.	Scarlet pimpernel
Anchusa azurea Mill.	Garden anchusa/bugloss
Anethum graveolens L.	Garten-Dill
Anthriscus cerefolum (L.) Hoffm.	Garden chervil
Apium graveolens L.	Celery/marsh parsley
Arctium lappa L.	Burdock
Armoracia rusticana G.Gaertn., B.Mey. & Scherb.	Common horse radish
Artemisia abrotanum L.	Southernwood/slovenwood
Artemisia absinthium L.	Worm-wood/absinthium
Artemisia dracunculus L.	Tarragon
Arum maculatum L.	Cuckoopint

Table S2. (*continued*).

**Botanical name**	**Common name**
Arum orientale M.Bieb.	Friar's cowl
Asparagus officinalis L.	Sparrow grass/asparagus
Asperula odorata L.	Sweet woodruff
Astragalus microcephalus Willd.	Astragalus
Astragalus strictifolius Boiss.	Milk vetch
Atropa bella-donna L.	Deadly nightshade
Atropa caucasica Kreyer	Caucasian belladonna
Barbarea vulgaris W.T.Aiton	Winter cress/yellow rocket
Berberis iberic Steven & Fisch. ex DC.	Georgian bar berry
Berberis orientalis C. Schneid.	Eastern barley
Berberis vulgaris L.	Bar berry
Betonica officinalis L.	Wood betony
Betula litwinowii (Doluch.) Ashburner & McAll.	-
Betula pendula Roth	Drooping birch/weeping birch
Betula pubescens Ehrh.	White birch/downy birch
Bifora radians M.Bieb.	Bifora
Brassica juncea (L.) Czern.	Brown mustard
Bryonia alba L.	Mandrake
Bryonia dioica (Jacq.) Tutin	Red bryony
Bunias orientalis L.	Hill mustard
Butomus umbellatus L.	Flowering-rush
Caltha palustris L.	Marsh-marigold
Caltha polypetala Hochst.	Giant Marsh Marigold
Calystegia sepium (L.) R.Br.	Hedge bindweed/bellbind
Capparis spinosa L.	Caper-bush/flinders rose
Capsella bursa-pastoris Medik.	Shepherd's purse/lady's purse
Carex brevicollis DC.	Sedge
Carum carvi L.	Cumin
Castanea sativa Mill.	Chestnut
Celtis caucasica Willd.	Caucasian hackberry/Caucasian nettle tree
Celtis glabrata Stev. ex Planch.	Sugar hackberry

Table S2. (*continued*).

Botanical name	Common name
Centaurea cyanus L.	Centaury/bull-weed/flame tree
Centaurea hajastana Tzvel.	Knapweed
Centaurium erythraea Rafn	Common centaury
Cerasus avium (L.) *Moench*	Sweet cherry/merry
Chelidonium majus L.	Ceylon cornel tree
Chenopodium album L.	Goosefoot/lamb's quarters
Chondrilla juncea L.	Skeleton weed/gum succory
Cichorium intybus L.	Succory/endive
Clematis vitalba L.	Old man's beard/traveller's joy
Cnicus benedictus L.	Blessed thistle/holy thistle
Colchicum speciosum Stev.	Meadow-saffron
Convallaria majalis L.	Lily-of-the-valley
Coriandrum sativum L.	Coriander
Cornus mas L.	Cornel
Coronilla varia L.	Crown vetch
Corylus avellana L.	Common hazel
Cotinus coggygria Scop.	Smoke tree
Cotoneaster integerrimus Medik.	Common cotoneaster
Cotoneaster melanocarpus Fisch. ex Loudon	Rockspray cotoneaster
Crataegus armena Pojark.	Armenian hawthorn
Crataegus orientalis (Mill.) M.Bieb.	Hawberry/may-tree
Crataegus pallasii Griseb.	Woodland hawthorn
Cuscuta epithymum L.	Thyme dodder
Cuscuta europaea L.	Great dodder
Cuscuta monogyna Vahl	Dodder
Daphne mezereum L.	Widow-wail/spurge
Datisca cannabina L.	False hemp/bastard-hemp
Datura stramonium L.	Thorn apple
Daucus carota L.	Carrots
Dictamnus caucasicus Fisch. ex Grossh.	Burning bush/dittany
Digitalis ferruginea L.	Rusty foxglove

Table S2. (*continued*).

Botanical name	Common name
Diphelypaea coccinea (M.Bieb.) Nicolson	-
Dipsacus strigosus Willd. ex Roem. & Schult.	Teasel
Dracocephalum moldavica L.	Moldavian dragonhead
Echinops sphaerocephalus L.	Hedgehog plant
Echium vulgare Brot.	Viper's-bugloss/blueweed
Elaeagnus angustifolia L.	Oil tree/oleaster
Elytrigia repens (L.) Nevski.	Coach grass/shelly grass
Ephedra distachya L.	Ephedra/sea grape
Equisetum arvense L.	Shave-grass/rough horse-tail
Eremurus spectabilis M.Bieb.	Eremurus
Eruca vesicaria (L.) Cav.	Garden rocket
Eryngium campestre L.	Eryngo/sea-holly
Euphrasia hirtella Jord. ex Reut.	Eyebright
Filipendula hexapetala Gilib.	Dropwort/meadowsweet
Filipendula ulmaria (L.) Maxim.	Dropwort
Foeniculum vulgare Mill.	Fennel
Fragaria vesca L.	Wild strawberry
Fragaria viridis Weston	Creamy strawberry
Frangula alnus Mill.	Alder buckthorn/black alder
Fumaria officinalis L.	Common fumitory
Fumaria schleicheri Soy.-Will.	Dark fumitory
Galega officinalis L.	Common goat's rue
Galium aparine L.	Cleavers/catchweed
Galium verum L.	Lady's bedstraw/yellow bedstraw
Gentiana cruciata L.	Star gentian
Geranium collinum Steph. ex Willd.	Crane's bill
Geum rivale L.	Water avens
Geum urbanum L.	Roof avens
Glechoma hederacea L.	Ground-ivy/wild snakeroot
Glycyrrhiza glabra L.	Licorice
Grossularia reclinata Mill.	Gooseberry

Table S2. (*continued*).

Botanical name	Common name
Gypsophila bicolor Grossh	Chalk plant/babies' breath
Hemerocallis fulva (L.) L.	Orange day-lily
Heracleum antasiaticum Manden.	Cow-parsnip
Heracleum transcaucasicum Manden.	Transcaucasian Hogweed
Herniaria glabra L.	Smooth rupturewort
Hippophae rhamnoides L.	Sea-buck thorn
Humulus lupulus L.	Common hop/bine
Hyoscyamus niger L.	Henbane
Hypericum alpestre subsp. Polygonifolium (Rupr.) Avet. & Takht.	Perfoliate St. John's wort
Hypericum eleonorae Jelen.	Armenian St. John's-wort
Hypericum perforatum L.	St. John's wort
Hyppophae rhamnoides L.	Sea buckthorn
Hyssopus angustifolius M.Bieb.	Hyssop
Inula helenium L.	Common inula/ horse elder
Juglans regia L.	European walnut
Juniperus oblonga M.Bieb.	Junipers/common juniper tree
Lactuca serriola L.	Prickly lettuce/ milk thistle
Lamium album L.	White dead-nettle
Lamium maculatum L.	Spotted dead-nettle
Laser trilobum Borkh. ex Gaertn.	Trilobate laserwort
Lathraea squamaria L.	Common toothwort
Lathyrus pratensis L.	Yellow pea/meadow pea/ meadow vetchling
Lathyrus tuberosus L.	Groundnut peavine/tuberous pea
Laurus nobilis L.	Bay laurel
Lemna minor L.	Duckweed
Leontodon hispidus L.	Bristly hawkbit/ rough hawkbit
Leonurus cardiaca L.	Motherwort
Lepidium latifolium L.	Pepper-grass/ pepperwort
Lepidium campestre (L.) W.T.Aiton	Field pepperwort
Lilium armenum (Miscz. ex Grossh.) Manden.	Armenian lily

Table S2. (*continued*).

**Botanical name**	**Common name**
Linaria vulgaris Mill.	Common toadflax
Lithospermum officinale L.	Gromwell
Lotus corniculatus L.	Common bird's-foot trefoil/birdsfoot deervetch
Lythrum salicaria L.	Purple loosestrife
Malus orientalis Uglitzk.	Caucasus apple
Malva neglecta Wallr.	Dwarf mallow
Malva sylvestris L.	Common mallow/round dock
Marrubium vulgare L.	Melic grass/mountain melic
Matricaria chamomilla L.	Chamamel
Matricaria matricarioides (Less.) Porter	Matricary
Melampyrum arvense L.	Cow-wheat
Melilotus officinalis (L.) Pall.	Melilot
Melissa officinalis L.	Lemon-balm/bee-balm
Mentha arvensis L.	Field mint/corn mint
Mentha longifolia (L.) L.	Horse mint
Mentha pulegium L.	Mint/pudding grass
Menyanthes trifoliata L.	Bean trefoil/bog bean/buckbean
Mespilus germanica L.	Medlar
Morus alba L.	White mulberry/common mulberry
Myosotis micrantha Pall. ex Lehm.	Forget-me-not
Myosotis sylvatica Hoffm.	Wood forget-me-not
Myosoton aquaticum Moench.	Water chickweed/giant chickweed
Nasturtium officinale R.Br.	Watercress/yellowcress
Nepeta grandiflora M.Bieb.	Catnip/catmint
Nigella sativa L.	Black caraway
Nymphaea alba L.	White water-lily
Ocimum basilicum L.	Basil/sweet basil
Ononis arvensis L.	Field restharrow
Onopordum acanthium L.	Cotton thistle
Orchis mascula L.	Early-purple orchid
Orchis palustris Jacq.	Jersey orchis

Table S2. (*continued*).

**Botanical name**	**Common name**
Origanum vulgare L.	Wild marjoram
Orobanche aegyptiaca Pers.	Egyptian broomrape
Orobanche lutea Baumg.	Species broomrape
Orobanche ramosa L.	Branched broomrape
Oxalis corniculata L.	Creeping woodsorrel/sleeping beauty
Oxalis acetosella L.	Wood sorrel
Padus racemosa (Lam.) Gilib.	Bird cherry
Papaver orientale L.	Poppy
Peganum harmala L.	Harmel/wild rue
Phlomis tuberosa L. Moench	Jerusalem sage
Phragmites australis (Cav.) Steud.	Common red grass
Physalis alkekengi L.	Bladder cherry/winter cherry
Pinus kochiana Klotzsch ex K. Koch	Pine
Pistacia atlantica Desf.	Pistachio tree
Pistacia mutica Fisch. Et C. A. Mey	Turpentine tree
Plantago major L.	Broad-leaved plantain/Plantain
Plantago lanceolata L.	Ribwort plantain/narrowleaf plantain
Polygala anatolica Boiss. et Heldr.	Milkwort
Polygonatum glaberrimum K. Koch.	Solomon’s seal
Polygonum aviculare L.	Knott-grass/knotweed
Polygonum carneum C.Koch.	Snakeweed
Polygonum hydropiper L.	Water pepper/biting knotweed
Polygonum persicaria L.	Lady´s thumb
Portulaca oleracea L.	Common purslane
Potentilla erecta (L.) Raeusch.	Tormentil
Poterium polygamum Waldst. et Kit.	Garden burnet
Primula veris L.	Cowslip primrose
Prunella vulgaris L.	Self-heal
Prunus armeniaca L.	Aprikot
Prunus divaricata Ledeb.	Cherry plum/myrobalan plum
Prunus spinosa L.	Blackthorn

Table S2. (*continued*).

**Botanical name**	**Common name**
Pulmonaria dacica Simonk.	Lungwort
Pulsatilla alban (Steven) Bercht. & J.Presl,	Passflower/bluemony
Punica granatum L.	Pomegranate
Pyrus caucasica Fed.	Pear
Quercus araxina (Trautv.) Grossh.	Unknown
Quercus iberica Stev.	Georgian oak
Quercus macranthera Fisch. & Mey. Ex Hohen.	Caucasian oak
Quercus robur L.	Pedunculate oak
Raphanus raphanistrum L.	White charlock/wild radisch
Reseda lutea L.	Wild mignonette
Rhamnus cathartica L.	Purging buckthorn
Rheum luteola L.	Dyeweed
Rheum palmatum L.	Rhubarb
Rhinanthus minor L.	Yellow rattle
Rhinanthus pectinatus (Behrend.) Vass.	Rattle-box
Rhus coriaria L.	Peacock flower
Ribes alpinum L.	Mountain currant
Ribes armenum Pojark. (*Ribes nigrum* L.)	Black currant
Ribes biebersteinii Berl. Ex Dc	Biberstein currants
Rosa canina L.	Dog-rose/heprose
Rosa corymbifera Borkh.	Spiny rose
Rosa sosnovskyana Tam.	Transcaucasian rose
Rosa spinosissima L.	Burnett-Rose
Rubia tinctorum L.	Madder
Rubus armeniacus Focke	Armenian blackberry
Rubus caesius L.	Tamarisk
Rubus idaeus L.	Raspberry
Rubus saxatilisn L.	Stone bramble
Rubus takhtadjanii Mulk.	Armenian raspberry
Rumex alpinus L.	Monk's-rhubarb
Rumex crispus L.	Curly dock/curled dock

Table S2. (*continued*).

Botanical name	Common name
Sagittaria sagittifolia L.	Old-world arrowhead
Salicornia europaea L.	Common glasswort/glasswort
Salix alba L.	Common willow/white willow
Salix caprea L.	Goat willow
Salix cinerea L.	Gray-leaved willow
Salvia officinalis L.	Sage/garden sage
Salvia sclarea L.	Clary/clary sage
Sambucus ebulus L.	Danewort/dwarf elder
Sambucus nigra L.	Boon tree
Sanguisorba officinalis L.	Burnet
Saponaria officinalis L.	Cartilage/soap
Satureja hortensis L.	Summer savory
Scorzonera latifolia (Fisch. et Mey.) DC.	Scorzonera/salsify
Sedum acre L.	Goldmoss stonecrop/wallpepper
Sedum album L.	Stonecrop
Senecio rhombifolia (Adam) Sch. Bip.	Groundsel
Sinapis arvensis L.	Charlock/wild mustard
Sisymbrium officinale (L.) Scop.	Hedge mustard/wild rocket
Solanum dulcamara L.	Bitter-sweet
Solanum nigrum L.	Black nightshade
Sophora japonica (L.) Schott	Japanese pagoda tree/pagoda tree
Sorbus aucuparia L.	Mountain ash
Sorbus hajastana Gabrieljan	Armenian rowan
Sorghum halepense (L.) Pers.	Johnson grass
Stachys palustris L.	Clown's woundwort/ marsh hedgenettle
Stellaria media (L.) Vill.	Starwort/chickweed
Symphytum asperum Lepech.	Prickly comfrey/rough comfrey
Tamus communis L.	Black bryony
Tanacetum coccineum (Willd.) Grierson	Pyrethrum/feverfew
Tanacetum vulgare L.	Common tansy
Taraxacum officinale (L.) Webb ex F.H.Wigg.	Bristle-fern

Table S2. (*continued*).

**Botanical name**	**Common name**
Taxus baccata L.	Common yew/European yew
Teucrium polium L.	Cat thyme/hulwort/mountain germander
Teucrium scordioides Schreb.	Wood germander
Thalictrum foetidum L.	Meadow-rue
Thalictrum minus L.	Lesser meadow-rue
Thymus collinus Bieb.	Hill thyme
Thymus kotschianus Boiss. Et Hohen.	Thyme
Thymus serpyllum L.	Wild thyme/mother-of-thyme
Thymus transcaucasicus Ronn.	Transcaucasian thyme
Tilia caucasica Rupr.	Caucasian linden
Tilia cordata Mill.	Small-leaved linden
Tribulus terrestris L.	Caltrop
Trifolium pratense L.	Red clover/cow clover/meadow clover
Trifolium repens L.	White clover/creeping trefoil/shamrock
Trigonella foenum-graecum L.	Fenugreek
Tussilago farfara L.	Cough-wort/horse nail
Typha angustifolia L.	Narrow-leaved cat´s-tail
Typha latifolia L.	Broad-leaved cat´s-tail/mace reed
Urtica dioica L.	Large nettle
Urtica urens L.	Small nettle/burning nettle
Vaccinium myrtillus L.	Bilberry/whortleberry
Valeriana officinalis L.	Valerian
Veratrum album L.	False hellebore
Veratrum lobelianum Bernh.	Lobel's Hemeritzia
Verbascum speciosum Schrad.	Mullein
Verbascum phlomoides L.	Orange mullein/woolly mullein
Verbena officinalis L.	Common vervain/common verbena
Veronica beccabunga L.	European speedwell/brooklime
Viburnum opulus L.	Marsh elder/common snowball
Viburnum lantana L.	Wayfarer/wayfaring tree
Vinca minor L.	Periwinkle

Table S2. (*continued*).

**Botanical name**	**Common name**
Viola odorata L.	Violet
Viola arvensis Murray	Field pansy
Viola tricolor L.	Wild pansy/Johnny Jump up
Viscum album L.	Common mistletoe/Viscin
Vitis sylvestris C. C. Gmel.	Woodland grape
Xanthium strumarium L.	Clotbur/cocklebur
Xeranthemum squarrosum Boiss.	Common immortelle
Ziziphus jujuba Mill.	Jujube
Zygophyllum fabago L.	Bean caper
